# Association of Maternal Stress and Social Support During Pregnancy With Growth Marks in Children’s Primary Tooth Enamel

**DOI:** 10.1001/jamanetworkopen.2021.29129

**Published:** 2021-11-09

**Authors:** Rebecca V. Mountain, Yiwen Zhu, Olivia R. Pickett, Alexandre A. Lussier, Jill M. Goldstein, Joshua L. Roffman, Felicitas B. Bidlack, Erin C. Dunn

**Affiliations:** 1 Psychiatric and Neurodevelopmental Genetics Unit, Center for Genomic Medicine, Massachusetts General Hospital, Boston; 2 Department of Psychiatry, Massachusetts General Hospital, Harvard Medical School, Boston; 3 Department of Epidemiology, Harvard T.H. Chan School of Public Health, Boston, Massachusetts; 4 Henry and Allison McCance Center for Brain Health, Massachusetts General Hospital, Boston; 5 Department of Obstetrics, Gynecology, and Reproductive Biology, Massachusetts General Hospital, Boston; 6 Innovation Center on Sex Differences in Medicine, Massachusetts General Hospital, Boston; 7 Forsyth Institute, Cambridge, Massachusetts; 8 Department of Developmental Biology, Harvard School of Dental Medicine, Boston, Massachusetts

## Abstract

**Importance:**

Exposure to maternal psychosocial stressors during the prenatal and perinatal periods can have major long-term mental health consequences for children. However, valid and inexpensive biomarkers are currently unavailable to identify children who have been exposed to psychosocial stress and the buffers of stress exposure.

**Objective:**

To assess whether a growth mark in tooth enamel, the neonatal line, is associated with exposure to prenatal and perinatal maternal psychosocial factors.

**Design, Setting, and Participants:**

This prospective cohort study used exfoliated primary canine teeth and epidemiological survey data from 70 children enrolled in the Avon Longitudinal Study of Parents and Children, a birth cohort based in Bristol, England. Exfoliated teeth were collected from children at 5 to 7 years of age. Data were collected from January 1, 1991, to December 31, 1998, and were analyzed from January 1, 2019, to August 10, 2021.

**Exposures:**

Four types of prenatal and perinatal maternal psychosocial factors were studied: stressful life events, psychopathological history, neighborhood disadvantage, and social support. Data were collected from mailed-in questionnaires completed during and shortly after pregnancy.

**Main Outcomes and Measures:**

Neonatal line width measured within 3 portions of the tooth crown (the cuspal, middle, and innermost third) in exfoliated primary canines.

**Results:**

A total of 70 children (34 of 70 [48.7%] male; 63 of 67 [94.0%] White) were studied. Most children were born full term (57 [83.8%]) and to mothers of typical child-bearing age (60 [88.2%]). Neonatal lines were wider in the canines of children born to mothers who self-reported severe lifetime depression (β = 3.35; 95% CI, 1.48-5.23; *P* = .001), any lifetime psychiatric problems (β = 2.66; 95% CI, 0.92-4.41; *P* = .003), or elevated anxiety or depressive symptoms at 32 weeks’ gestation (β = 2.29; 95% CI, 0.38-4.20; *P* = .02). By contrast, neonatal lines were narrower in children born to mothers who self-reported high social support shortly after birth (β = −2.04; 95% CI, −3.70 to −0.38; *P* = .02). The magnitude of these associations was large, up to 1.2 SD unit differences, and persisted after adjusting for other risk factors.

**Conclusions and Relevance:**

In this cohort study, neonatal line width was associated with exposure to maternal perinatal psychosocial factors. Replication and validation of these findings can further evaluate teeth as possible new biomarkers.

## Key Points

**Question:**

Can exfoliated primary teeth be used to identify children exposed to psychosocial risk and protective factors during their prenatal and perinatal life?

**Findings:**

This cohort study of 70 children from the Avon Longitudinal Study of Parents and Children birth cohort found evidence that teeth may record early-life events. Children exposed to prenatal maternal depression or anxiety had wider neonatal lines, a marker of enamel growth, and children with perinatal maternal social support had narrower neonatal lines.

**Meaning:**

Exfoliated primary teeth, specifically neonatal line width, may be associated with prenatal and perinatal life experiences.

## Teaser

This cohort study examines whether the teeth of children exposed to common prenatal and perinatal maternal stressors have wider neonatal lines, a marker of tooth enamel growth, compared with children without such exposure.

## Introduction

Children’s exposure to prenatal and perinatal maternal psychosocial stressors, such as psychopathological symptoms,^1,2,3^ stressful life events,^4,5^ and neighborhood disadvantage,^6,7^ can impact brain health across the life course. In addition to nearly doubling the risk of a mental health disorder,^8,9^ maternal psychosocial stress can become biologically embedded^10,11^ in children, resulting in lifelong physiological and neurobiological disruptions.^7,12,13,14^ Conversely, increased maternal social support^15^ is a known protective factor, associated with reduced inflammation in offspring during the first year of life,^16^ and fewer internalizing and externalizing symptoms across development.^17^ Collectively, these empirical findings are consistent with the developmental origins of health and disease and prenatal programming hypotheses, which propose that the intrauterine environment shapes development and risk of disease across the life course.^2,18,19,20,21^ These findings also underscore the importance of characterizing maternal stressors and social support to better understand their associations with pediatric mental health.^22^

However, several measurement challenges hinder these goals. For example, detailed prenatal medical records are often unavailable, causing studies to rely on retrospective maternal self-reports, which may be susceptible to memory and recall biases.^23,24,25^ Retrospective reports may also lack information about the intensity, duration, and timing of psychosocial exposures, which, if available, could guide tailored preventive efforts. Although prospective reports provide more detailed measurements of various exposures, they are subjective and often costly, invasive, and time-consuming. Thus, there is a need for novel measurement tools that can objectively, as well as inexpensively and noninvasively, provide information (beyond self-reports) about children’s exposure to prenatal maternal stress and social support.

Addressing this need for new tools, Davis et al^26^ recently proposed the TEETH (Teeth Encoding Experiences and Transforming Health) conceptual model for the use of teeth as biomarkers of early-life adversity and subsequent mental health risk. Building on this model, we investigated the extent to which measures of maternal psychosocial stress, a major form of early-life adversity, are associated with tooth-based markers, specifically the neonatal line (NNL). We also sought to expand the model developed by Davis et al^26^ to explore how protective factors, specifically social support, may be likewise captured by tooth-based markers.

**Figure 1.**
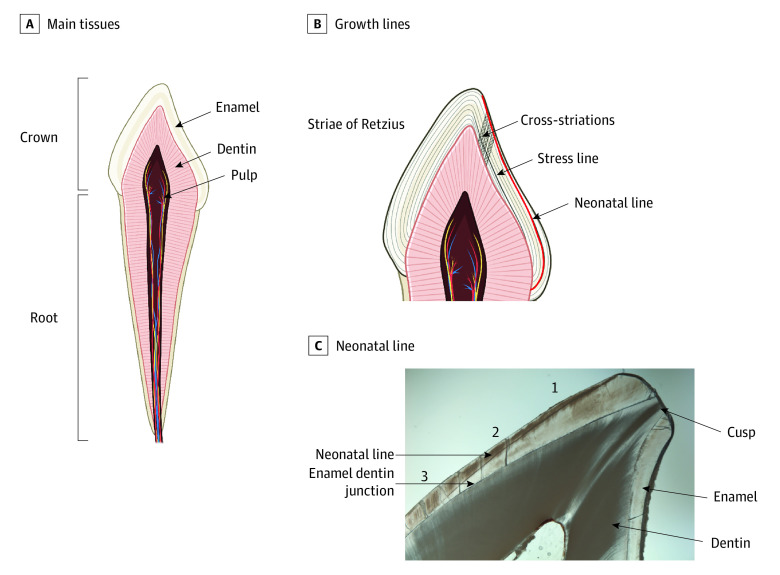
Primary Tooth Development and the Neonatal Line A, Primary teeth are composed of 3 main tissues: enamel, dentin, and pulp. Enamel is formed by cells called ameloblasts, which operate in a circadianlike process to lay down the enamel matrix in incremental layers; this incremental growth process is permanently recorded in enamel and dentin through a series of growth lines, which can be observed in longitudinal cross-sections of the tooth using light microscopy.^27,28^ B, Human teeth show daily growth lines, called cross-striations, which appear between longer period growth lines^27^ called striae of Retzius^28^ that correspond to roughly weekly growth. When an insult or disruption occurs during enamel or dentin formation, the growth mark may appear wider or darker; these more pronounced growth marks are referred to stress lines^29,30^ (or accentuated lines). C, The neonatal line is 1 of the most prominent stress lines, present in approximately 90% of primary teeth and 10% of permanent first molars.^31^ In this study, the neonatal line width was measured 3 times at 3 locations along the enamel prism: (1) the cuspal third or third closest to the enamel surface (referred to as the cusp), (2) the middle third, and (3) the third closest to the enamel-dentin junction, the point where the enamel and dentin meet.

The NNL has been used in anthropology for decades to distinguish between prenatal and postnatal enamel and characterize the overall stress of the birth process (Figure 1).^27,28,29,30,31^ One of the most prominent stress lines in teeth, the NNL has previously been investigated in conjunction with certain prenatal and perinatal factors, including maternal health and delivery characteristics^31,32,33,34,35^ (eTable 1 in the Supplement). Notably, stressful prenatal and perinatal conditions,^31,32,33,34,35^ such as complicated delivery, longer duration of delivery, and preterm births, have been associated with wider NNLs.

Although the NNL is an established anthropological marker of stressful gestational events, it remains unknown whether it could act as a biomarker of prenatal and perinatal psychosocial stress in modern pediatric populations. A handful of nonhuman primate studies suggest exposure to psychosocial stressors during early life, such as separation from the mother^36^ or social group^37^ or death of a sibling,^38^ may coincide with the presence of postnatal stress lines. However, this possibility has yet to be systematically investigated in humans. We describe what is, to our knowledge, the first study to characterize the association between maternal psychosocial factors and any tooth-based measure in living humans.

In this study, we tested the hypothesis that children exposed to common prenatal and perinatal maternal stressors (stressful life events, maternal psychopathological symptoms and diagnoses, and neighborhood disadvantage) display wider NNLs—indicative of more stressful conditions—than unexposed children. We also tested the hypothesis that children exposed to protective effects (greater social support for the mother) display narrower NNLs, even after controlling for physiological confounders.

## Methods

### Sample and Procedures

We analyzed 70 primary teeth collected from 70 children enrolled in the Avon Longitudinal Study of Parents and Children (ALSPAC), a prospective, population-based birth cohort in Bristol, England, beginning in the 1990s and designed to increase understanding of the genetic and environmental factors associated with disease across the life course (eMethods in the Supplement). Parents donated naturally exfoliated primary canine teeth when the children were between 5 and 7 years of age.^39^ Canines are easier to analyze because of their lower levels of wear, thicker enamel, and extended duration of crown formation.^40^ The sample size of 70 teeth is larger than those in previous studies^33,34,41,42,43^ of perinatal exposures on NNL width, which ranged from 11 to 65 teeth. The children in the analytic sample had sociodemographic features similar to the full ALSPAC sample except that they were more likely to come from families with higher socioeconomic positions (eTable 2 in the Supplement). The ALSPAC website contains details of all data available through a fully searchable data dictionary and variable search tool.^39,44^ Ethical approval for the study was obtained from the ALSPAC Ethics and Law Committee and the local research ethics committees. Written consent for all biological samples was collected in accordance with the Human Tissue Act,^45^ and all data were deidentified. Data analyzed in the current study were collected from January 1, 1991, to December 31, 1998, and were analyzed from January 1, 2019, to August 10, 2021. This study followed the Strengthening the Reporting of Observational Studies in Epidemiology (STROBE) reporting guideline.

### Measures

#### Neonatal Line Width

We analyzed 3 mean measures of NNL width derived by Hassett et al^40^ from longitudinal cross-sections imaged in widefield light microscopy using polarized light at ×2, ×20, and ×40 magnification with an VS-120 slide scanning system (Olympus Corp). Images were analyzed using ArcGIS software (ESRI), and the NNL was identified based on its position and appearance in the tooth crown, using established protocols.^46,47^ The mean value of 3 measurements of NNL width was obtained at 3 locations along its extension through the tooth crown: within the cuspal third, the middle third in lateral enamel, and the cervical third of the tooth crown, where the line is closest to the enamel dentine junction (EDJ)^40^ (Figure 1). All measurements were made on the enamel prism path from the EDJ to the enamel surface. Additional details on these measurements and the techniques applied are available in the study by Hassett et al.^40^

#### Maternal Psychosocial Factors

We examined 4 types of prenatal and perinatal maternal psychosocial factors: prenatal stressful life events, psychopathological history and symptoms, neighborhood disadvantage, and prenatal and perinatal social support. These factors were chosen because they represent prominent determinants of child health during the prenatal and perinatal periods.^8,15,48,49,50,51^ Data were self-reported from mailed-in questionnaires, which mothers completed during and shortly after pregnancy (Table 1; eAppendix in the Supplement).^52,53,54,55,56,57^

**Table 1.** Summary of Maternal Psychosocial Factors Examined in This Study

Measure	Period	Definition		
		Instrument(s)	Time point(s)	Importance
**Stressful life events**				
Partner emotional cruelty	Prenatal and perinatal	5 Items corresponding to these 2 sets of domains, taken from a 42-item stressful life events inventory	18 wk of Gestation and 8 wk after birth	Items captured experiences of interpersonal stress and complemented our focus on social support
Loss of a family member or friend	Prenatal and perinatal			
**Psychopathological history**				
Severe lifetime depression history	Prenatal	Report on history of 24 medical and psychiatric conditions	12 wk of Gestation	Items accounted for a possible latent trait of psychopathological history that complemented prospective survey measure
Any lifetime psychiatric problem	Prenatal			
Maternal depression or anxiety at 18 gestational weeks	Prenatal	The Crown-Crisp Experiential Index and the Edinburgh Postnatal Depression Scale^52,53,54^; presence of psychopathological symptoms was determined using previously established thresholds^53,55,56^	18 and 32 wk of gestation	Items accounted for changes in the time-varying state of maternal psychopathological symptoms during pregnancy rather than lifetime exposure
Maternal depression or anxiety at 32 gestational weeks	Prenatal			
**Neighborhood disadvantage**				
Neighborhood disadvantage	Prenatal	2 Measures composed of 10 items in total that asked mothers to indicate the degree to which they were concerned about safety and their impression of the neighborhood environment	8 wk of Gestation	Items captured socioeconomic disadvantage at the neighborhood level
**Social support**				
Social support at 12 gestational weeks	Prenatal	A 10-item questionnaire created by the ALSPAC team to measure perceived levels of social support in both the prenatal and perinatal periods^57^	12 wk of Gestation and 8 wk post partum	Items provided insights into protective factors, which complemented the analysis on stress
Social support at 8 wk post partum	Perinatal			

Abbreviation: ALSPAC, Avon Longitudinal Study of Parents and Children.

Race and ethnicity were measured as a binary variable (White vs non-White) in the study because the study sample in Avon, UK, was homogeneous, with predominantly White participants. While we recognize the importance of reporting racial and ethnic differences, a more refined categorization was not analytically feasible given our limited sample size.

### Statistical Analysis

We first performed univariate and bivariate analyses on all variables. We estimated correlations among binary psychosocial factors using tetrachoric correlations and correlations among continuous measures of NNL width using the Pearson *r*.

To estimate associations between psychosocial factors and NNL width, we adopted an approach that balanced our interest in reducing the number of tests performed and ensuring the parsimony of regression models; this principled approach was necessary given the study’s limited sample size. We first performed simple linear regression to obtain estimates for each of the 4 types of psychosocial factors (9 measures in total), unadjusted for covariates. Psychosocial factors with at least a nominal association (2-sided *P* < .05) with 1 or more of the NNL width measures in the unadjusted analysis were carried forward to a multiple linear regression model, where we further adjusted for covariates (eMethods in the Supplement). To account for multiple testing, we present false discovery rate–adjusted *P* values alongside standard *P* values (2-sided *P* < .05).^58^

We also performed 2 sets of sensitivity analyses (eMethods in the Supplement). First, because maternal genetic liability for depression could be associated with maternal psychopathological symptoms or history and potentially tooth-based markers, we also performed an analysis controlling for maternal polygenic risk scores for depression in the subsample of children with maternal genotype data available (n = 54). Second, we performed a mutually adjusted regression model to evaluate the effects of significant psychosocial factors simultaneously. All analyses were performed using R software, version 3.5.2 (R Project for Statistical Computing).^59^

## Results

### Sample Characteristics

A total of 70 children (34 of 70 [48.7%] male; 63 of 67 [94.0%] White; 4 of 67 [6.0%] non-White) were studied. Most children were born full term (57 of 68 children [83.8%]) and to mothers of typical child-bearing age (60 of 68 [88.2%]) (eTable 2 in the Supplement). Reports of psychosocial stress were common, ranging from 4 of 61 (6.6%) (neighborhood disadvantage) to 17 of 67 (25.4%) (loss of friend or family during pregnancy). A total of 15 of 70 children (21.4%) were exposed to maternal psychopathological symptoms or prior diagnoses. Although only 3 of 62 mothers (4.8%) reported high levels of social support in the first trimester, this number increased to 9 of 62 (14.5%) shortly after birth. Correlations between psychosocial factors are reported in eFigure 1 in the Supplement.

Primary canines came from each of the 4 dental quadrants (18 in the upper right, 13 in the upper left, 22 in the lower left, and 17 in the lower right). The mean (SD) NNL width was highest in the cuspal enamel portion (11.83 [4.97] μm) followed by the middle portion (9.87 [3.8] μm) and the EDJ portion (7.34 [2.46] μm) (eFigure 2 in the Supplement). Measures were correlated within each portion but distinct across portions, suggesting that tooth-based markers at the 3 portions may capture different biological signatures (eFigures 3 and 4 in the Supplement).

### Associations Between Maternal Psychosocial Factors and Tooth-Based Markers

**Figure 2.**
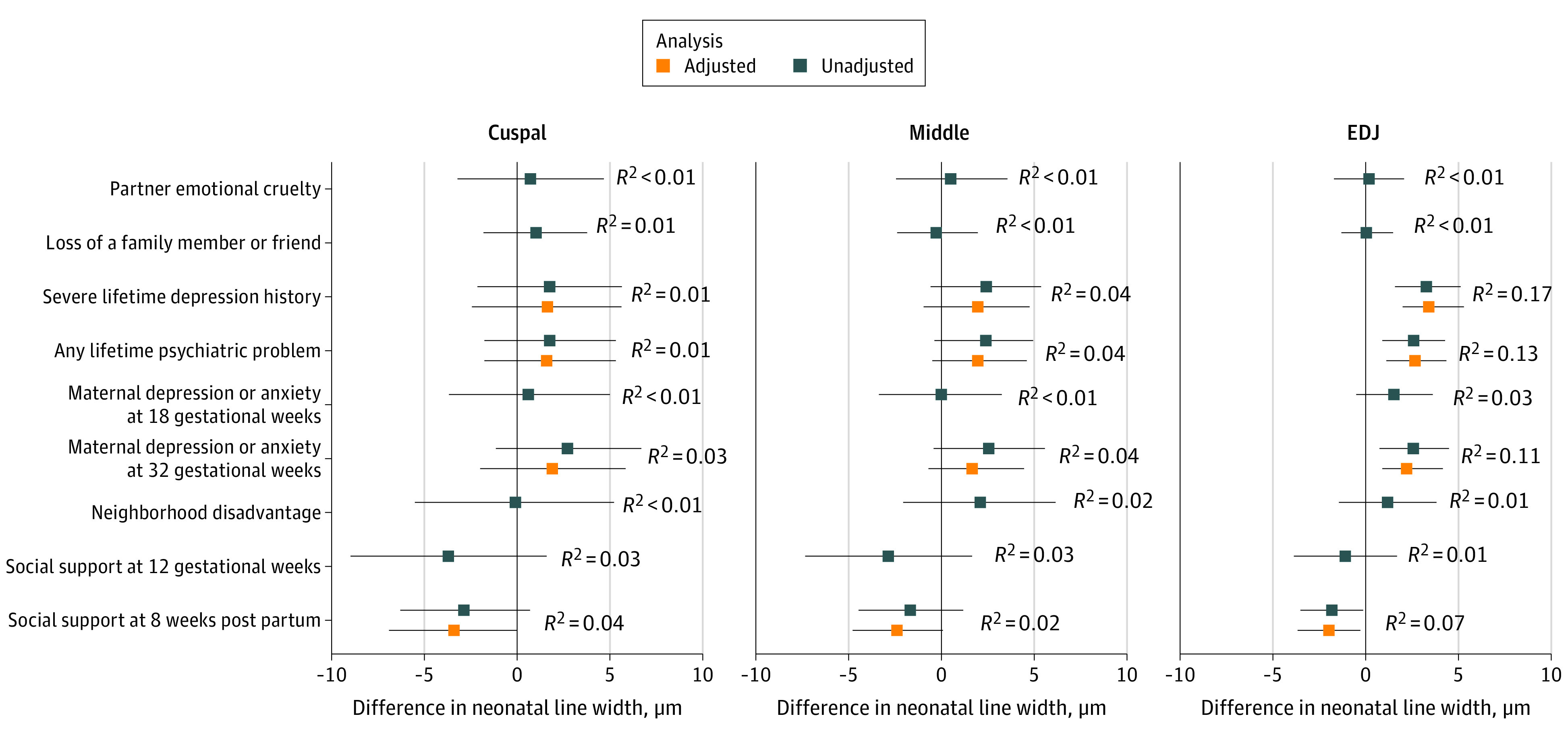
Unadjusted and Adjusted Associations Between Perinatal Maternal Psychosocial Factors and Mean Neonatal Line Widths Measured at the Cuspal, Middle, and Enamel-Dentine Junction (EDJ) Sections Psychosocial factors showing a nominal association (*P* < .05) with 1 or more of the neonatal line width measures in the unadjusted analysis were examined in the adjusted analyses. Error bars indicate 95% CIs.

The unadjusted analyses indicated no evidence of an association between exposure to psychosocial factors and cuspal or middle NNL widths. However, exposures to 4 psychosocial factors were associated with NNL width near its intersection with the EDJ (Figure 2; eTable 3 in the Supplement), with each psychosocial factor (when tested separately) being associated with 7% to 17% of the variation. Three of those factors pertained to maternal psychopathological history, specifically, severe lifetime depression history, any lifetime psychiatric problem, and maternal depression or anxiety at 32 weeks’ gestation. Children born to mothers who self-reported these problems had wider mean NNLs in the EDJ portion compared with children who were unexposed (past depression: β = 3.31; 95% CI, 1.50-5.12; *P* < .001; psychiatric problems: β = 2.57; 95% CI, 0.91-4.24; *P* = .003; anxiety or depression: β = 2.62; 95% CI, 0.74-4.50; *P* = .007). Notably, severe lifetime depression and any lifetime psychiatric problem were strongly correlated (tetrachoric *r* = 0.98). We report both associations for completeness, enabling future studies (with measures of psychiatric history or depression) to directly compare their findings to ours. In the current study, we focused our interpretation of results on exposure to severe depression because it constituted 78% of the reported exposure to psychiatric problems in the analytic sample. The fourth factor associated with NNL width was high social support shortly after birth. The NNL was narrower in children of mothers who self-reported this factor when compared with children born to mothers without high support (β = −1.80; 95% CI, −3.49 to −0.12; *P* = .04) (Figure 2; eTable 3 in the Supplement).

In adjusted analyses, we controlled for 3 perinatal risk factors associated with NNL width in preliminary analyses: iron supplements during pregnancy, prepregnancy obesity, and gestational age (eTables 4-6 in the Supplement). The significant associations identified in unadjusted analyses (Figure 2) persisted in adjusted analyses for all 4 psychosocial factors (Table 2). Specifically, children born to mothers who self-reported having severe lifetime depression history had wider NNLs by 3.4 μm compared with children whose mothers were unexposed (95% CI, 1.48-5.23; *P* < .001), an estimate close to 1.5 times the SD of the EDJ measure. Similarly, children of mothers with any lifetime psychiatric problem had NNL widths 2.7 μm higher than children of mothers without any psychiatric history (95% CI, 0.92-4.41; *P* = .003). Children exposed to high maternal depression or anxiety symptoms at 32 weeks’ gestation (as opposed to lifetime exposure) had a wider measure of 2.3 μm in the EDJ portion of the NNL (95% CI, 0.38-4.20; *P* = .02). By contrast, being born to mothers with high social support shortly after birth was associated with a 2-μm narrower measure in the NNL width in the EDJ portion (95% CI, −3.70 to −0.38; *P* = .02) after covariate adjustment. A suggestive association between exposure to high social support and NNL widths in the outer enamel cuspal portion also emerged (β = −3.48; 95% CI, −6.92 to −0.04; *P* = .048). After correcting for testing 4 psychosocial factors in 3 portions of the NNL, associations between the following 2 psychosocial factors and NNL widths in the EDJ portion persisted: severe lifetime depression history (false discovery rate–adjusted *P* = .01) and any lifetime psychiatric problem false discovery rate–adjusted *P* = .02).

**Table 2.** Adjusted Associations Between Maternal Psychosocial Factors During Pregnancy and Neonatal Line Widths Measured at the Cuspal, Midcrown, and EDJ Adjacent Portions^a^

Exposure	β (SE) [95% CI]	*P* value	FDR-adjusted *P* value	Model overall *R*^2^
**Cuspal portion (mean [SD], 11.83 [4.97] μm)**				
Severe lifetime depression history	1.59 (2.03) [−2.47 to 5.64]	.44	.44	0.11
Any lifetime psychiatric problem	1.59 (1.84) [−2.09 to 5.27]	.39	.43	0.11
Maternal depression or anxiety, 32 gestational weeks	1.90 (1.96) [−2.02 to 5.82]	.34	.40	0.11
High social support, 8 wk post partum	−3.48 (1.72) [−6.92 to −0.04]	.048	.11	0.16
**Middle portion (mean [SD], 9.87 [3.8] μm)**				
Severe lifetime depression history	1.93 (1.47) [−1.00 to 4.86]	.19	.29	0.22
Any lifetime psychiatric problem	2.01 (1.33) [−0.64 to 4.66]	.13	.23	0.23
Maternal depression or anxiety, 32 gestational weeks	1.64 (1.43) [−1.21 to 4.49]	.25	.34	0.22
High social support, 8 wk post partum	−2.36 (1.23) [−4.83 to 0.10]	.06	.12	0.32
**EDJ portion (mean [SD], 7.34 [2.46] μm)**				
Severe lifetime depression history	3.35 (0.94) [1.48 to 5.23]	<.001	.01	0.24
Any lifetime psychiatric problem	2.66 (0.87) [0.92 to 4.41]	.003	.02	0.20
Maternal depression or anxiety, 32 gestational weeks	2.29 (0.96) [0.38 to 4.20]	.02	.06	0.16
High social support, 8 wk post partum	−2.04 (0.83) [−3.70 to −0.38]	.02	.06	0.18

Abbreviations: EDJ, enamel-dentine junction; FDR, false discovery rate.

^a^ Mean (SD) of each neonatal line portion are noted in the section headers to provide a reference for interpreting the magnitude of effect estimates. In these adjusted models, the following covariates were included: gestational age (continuous or weeks), maternal obesity before pregnancy (with 0 indicating a body mass index <30 and 1 indicating a BMI of 30 or greater [calculated as weight in kilograms divided by height in meters squared]), and maternal iron supplement during pregnancy (with 0 indicating did not take iron supplement and 1 indicating took iron supplement). These maternal or birth-related factors were selected as covariates because they were associated with the neonatal line width in bivariate analyses (eMethods in the Supplement).

Associations between psychosocial factors and NNL width at the EDJ generally persisted after adjusting for mother’s polygenic risk score for depression, although some estimates changed slightly (eTable 7 and eFigure 6 in the Supplement). Associations between maternal psychosocial factors and covariates are given in eTable 8 in the Supplement. Mutually adjusted analysis suggests that severe lifetime depression history and high social support post partum were still associated with NNL width at the EDJ portion (eTable 9 in the Supplement).

## Discussion

The main finding of this cohort study is that prenatal and early postnatal psychosocial exposures, both stressful and protective in nature, show evidence of biological embedding in teeth. Children exposed to prenatal maternal depression or anxiety and children of mothers with a history of severe depression or psychiatric problems had wider NNLs than unexposed children. One of the factors most strongly associated with a protective effect against depression, social support,^15^ was also associated with narrower NNL width. Each psychosocial factor explained a substantial proportion of the variation in the tooth-based markers. These associations persisted after controlling for multiple perinatal factors. The magnitude of associations was comparable to known perinatal factors associated with NNL widths, such as gestational age^31,40^ or maternal obesity.^40^ To our knowledge, this is the first study to demonstrate an association between maternal psychosocial stressors and child tooth-based measures, as proposed in the TEETH model by Davis et al.^26^

We found strong associations between elevated maternal depressive and anxiety symptoms at 32 weeks’ gestation and NNL width. However, we did not observe associations with the same maternal measures earlier in pregnancy (at 18 weeks’ gestation). What might explain this discrepancy? One possibility supported by dental and anthropological research is that the NNL, unlike other prenatal stress lines, forms and reflects experiences at the time of birth and shortly thereafter.^34,47^ Therefore, earlier (vs later) maternal exposures of time-varying symptoms (or state-level psychopathological markers) may have no associations with NNL width.

However, we identified strong associations between NNL width and self-reported maternal lifetime history of depression. Although it might seem to contradict the absence of association between depression or anxiety at 18 weeks’ gestation and NNL width, several different factors may explain the seeming inconsistency. First, NNLs could potentially reveal severe prior or long-term exposures that shape the uterine environment beyond moderate levels of symptoms captured during pregnancy. The severe preexisting vulnerabilities or a latent trait of psychopathological symptoms or history is likely distinct from time-varying (or state) symptoms, such as anxiety specifically related to pregnancy.^60^ As discussed previously, time-varying symptoms that do not persist in duration may not affect the NNL, but this lack of association does not preclude an association between severe lifetime depression history and the NNL. Second, because the sample size of the current study was small compared with other epidemiological studies,^55,61^ we were not statistically powered to detect small effects (eResults and eFigure 5 in the Supplement provide an a priori power analysis). Thus, it is plausible that earlier symptoms had an association with NNL width, but we were unable to observe that difference.

Notably, most of the signal identified came from the EDJ portion of the NNL, potentially suggesting time-dependent associations with maternal psychosocial stress. This result might be due to a confluence of factors related to the process of crown extension (the rate at which the tooth crown increases in height during development) and daily secretion rates (quantity of enamel produced by ameloblasts each day) specifically for canines, the analyzed tooth type. Exposures that occurred during stages of crown extension and enamel apposition may be more visibly encoded in the inner enamel or the EDJ portion of the NNL, whereas exposures that occurred during enamel maturation could more strongly affect the outer portions of the NNL. Crown formation and extension begin at the cusp of the tooth and proceed toward the cervical margin, where the enamel meets the root.^62^ The NNL and other long-period growth lines in enamel track the process of enamel matrix apposition, whereas their intersection with the EDJ indicates the rate of crown extension. During the incremental stages of enamel matrix apposition, the inner portion of the NNL, closest to the EDJ, is formed at a slower rate than the outer portions because of the decrease of crown extension rate from cuspal toward cervical and slower daily secretion rate of inner enamel.^63^ The slower pace of growth could incur higher susceptibility and therefore encode concurrent exposures more visibly. Furthermore, in canines, approximately 22% to 33% of the tooth crown is formed before birth, with the remaining 78% to 67% of the crown formed after birth.^63,64^

In our study, maternal psychosocial stress and the associated physiological signatures of stress during pregnancy were before or concurrent with the process of tooth crown formation. Therefore, it is unsurprising that we found these exposures were more consistently associated with differences in the NNL widths in the EDJ portion. In contrast, the process of enamel maturation starts after full enamel thickness is reached and is based on diffusion and exchange processes from the crown surface throughout the entire thickness of enamel. Events that occurred during the process of maturation might therefore be more visible in the outer portion of enamel and more attenuated in the inner portion of enamel. Our results further suggest social support had consistent associations across the NNL, potentially indicating that its effects were present throughout both stages of enamel formation and maturation. We hope future studies will combine epidemiological data with analyses of growth marks in different tooth types to elucidate whether and how the timing and type of perinatal exposures are captured during different stages of tooth formation and along different locations of the NNL.

There are several possible mechanisms through which maternal psychosocial stress may become biologically embedded in offspring’s teeth. For instance, maternal cortisol responses may influence enamel formation via insulinlike growth factors (IGFs).^65,66,67^ Cortisol increases following chronic psychosocial stress, decreasing IGF production in the hard tissues.^65^ Both IGF-1 and IGF-2 are involved in amelogenesis and positively associated with enamel production.^66^ Increased maternal cortisol production, as a result of psychosocial stress, may reduce IGF production in offspring. Higher maternal cortisol levels could likewise decrease amelogenesis during the perinatal period and result in a wider NNL. Conversely, enhanced social support may decrease maternal cortisol levels,^68^ possibly producing an inverse effect from psychosocial stress and therefore resulting in a narrower NNL. Few studies have examined the link between cortisol and tooth development; however, Boyce et al^67^ detected an association between cortisol reactivity and enamel measures in kindergarten-aged children. Mechanistic work on the biological embedding of adversity has also emphasized the potential role of inflammatory cytokines and epigenetics in shaping neurodevelopment,^69,70^ which may likewise impact tooth development. As such, future work should include additional maternal and child biospecimens to test these possible mechanisms.

Should these results be replicated in larger samples, they could have important implications for future intervention programs. Children naturally begin exfoliating teeth around 6 years of age.^71^ With more than half of mental health disorders diagnosed by early adolescence,^72^ early intervention around this time is critical and can have lifelong benefits. Children’s exfoliated teeth could be collected from pediatricians or dentists during routine checkups and sent to specialized laboratories for analysis. As is commonly done with other biospecimens during annual physical examinations, these teeth could be examined to detect adverse exposures that would be otherwise difficult to assess. In turn, the results could help identify children at risk and direct them toward evidence-based intervention programs, long before the onset of mental health symptoms.

### Limitations

This study has several limitations. First, although our sample size was large enough to detect associations with some primary exposures, it was smaller than typical epidemiological studies. Potential bias arising from small samples must be considered when interpreting results.^73^ Nonetheless, this sample size is larger than most studies^33,34,41,42,43^ exploring the association of perinatal exposures with the NNL. Second, given the sample size, we were unable to evaluate interactions among different maternal psychosocial factors; these inquiries remain important goals of future studies. Furthermore, we did not have the statistical power to test for differential effects of offspring sex on NNL morphology. Future studies should examine sex differences given the large body of work demonstrating varying effects of prenatal stress between male and female offspring, particularly in relation to the timing of exposure.^22,74^ Third, our measures of social support lacked granularity, particularly around the time of delivery. Additional analyses, such as partner support during labor and delivery, would help elucidate the association between protective factors and tooth-based measures. In addition, the lack of specificity on the characteristics of exposures, such as the timing of psychopathological symptom onset or infrequency of prospective reports, limited our ability to precisely interpret any time-dependent association between exposures and the NNL. Furthermore, the sparsity of available genetic data restricted our ability to investigate the potential genetic confounding of exposures to maternal psychopathological symptoms or history and tooth development. Fourth, the ALSPAC sample is predominantly well educated, White, and of high socioeconomic status, limiting the generalizability of our results. However, as reported in previous studies,^55,75^ the prevalence of exposure to childhood adversity in ALSPAC is comparable to estimates in other nationally representative samples.^76,77^ Although extreme adverse experiences may not have been captured in this population-based sample, our findings showcased the potential use of tooth-based markers in recording common psychosocial exposures.

## Conclusions

The findings of this cohort study suggest that perinatal psychosocial factors may show novel associations with the NNL. Future studies should attempt to replicate and validate this finding and investigate the links between tooth-based characteristics and child health outcomes. Such research could lay the groundwork for targeted intervention strategies to identify at-risk children and prevent future mental health disorders years before the onset of symptoms.

## Notes

Supplement. **eMethods.** Supplemental Methods **eResults.** Supplemental Results **eTable 1.** Perinatal Exposures Previously Investigated in Association With Neonatal Line Width: Results From 11 Studies **eTable 2.** Characteristics of the Analytic Sample (n=70) and Comparison to the ALSPAC Full Sample (n=14 901) **eTable 3.** Unadjusted Associations Between Perinatal Maternal Psychosocial Factors and Average Neonatal Line Widths Measured At The Cuspal, Middle, and Enamel-Dentine Junction (EDJ) Sections **eTable 4.** Unadjusted Associations Between Previously Examined Perinatal Factors and Neonatal Line Widths at the Cuspal Portion **eTable 5.** Unadjusted Associations Between Previously Examined Perinatal Factors and Neonatal Line Widths at the Middle Portion **eTable 6.** Unadjusted Associations Between Previously Examined Perinatal Factors and Neonatal Line Widths at the Enamel-Dentine Junction (EDJ) Portion **eTable 7.** Adjusted Associations Between Maternal Psychosocial Factors During Pregnancy and Neonatal Line Widths, Measured at the Cuspal, Mid-Crown, and Enamel-Dentine Junction (EDJ) Adjacent Portions, Controlling for Maternal Polygenic Risk Score (PRS) for Major Depressive Disorder **eTable 8.** Associations Between Maternal Psychosocial Factors and Three Covariates Included in the Current Study: Gestational Age, Maternal Prepregnancy Obesity, and Maternal Iron Supplements During Pregnancy **eTable 9.** Mutually Adjusted Associations Between Three Maternal Psychosocial Factors During Pregnancy and Neonatal Line Widths, Measured at the Cuspal, Mid-Crown, and Enamel-Dentine Junction (EDJ) Adjacent Portions **eFigure 1.** Correlations Between Measures of Maternal Psychosocial Factors **eFigure 2.** Distributions of Neonatal Line Width Measures **eFigure 3.** Correlations Between Measures of Neonatal Line Width **eFigure 4.** Tooth Position and the Neonatal Line Width Across Individuals **eFigure 5.** A Priori Power Analysis **eFigure 6.** Comparison of Associations Between Maternal Psychosocial Factors During Pregnancy and Neonatal Line Widths Before and After Controlling for Maternal Polygenic Risk Score (PRS) for Major Depressive Disorder
eReferences
