# Supplementary material for: Association of Maternal Stress and Social Support During Pregnancy With Growth Marks in Children’s Primary Tooth Enamel
Source: JAMA Netw Open. 2021 Nov 9;4(11):e2129129. doi: 10.1001/jamanetworkopen.2021.29129 (PMC8579236; doi:10.1001/jamanetworkopen.2021.29129)
Supplement: Supplement. — eMethods. Supplemental Methods eResults. Supplemental Results eTable 1. Perinatal Exposures Previously Investigated in Association With Neonatal Line Width: Results From 11 Studies eTable 2. Characteristics of the Analytic Sample (n=70) and Comparison to the ALSPAC Full Sample (n=14 901) eTable 3. Unadjusted Associations Between Perinatal Maternal Psychosocial Factors and Average Neonatal Line Widths Measured At The Cuspal, Middle, and Enamel-Dentine Junction (EDJ) Sections eTable 4. Unadjusted Associations Between Previously Examined Perinatal Factors and Neonatal Line Widths at the Cuspal Portion eTable 5. Unadjusted Associations Between Previously Examined Perinatal Factors and Neonatal Line Widths at the Middle Portion eTable 6. Unadjusted Associations Between Previously Examined Perinatal Factors and Neonatal Line Widths at the Enamel-Dentine Junction (EDJ) Portion eTable 7. Adjusted Associations Between Maternal Psychosocial Factors During Pregnancy and Neonatal Line Widths, Measured at the Cuspal, Mid-Crown, and Enamel-Dentine Junction (EDJ) Adjacent Portions, Controlling for Maternal Polygenic Risk Score (PRS) for Major Depressive Disorder eTable 8. Associations Between Maternal Psychosocial Factors and Three Covariates Included in the Current Study: Gestational Age, Maternal Prepregnancy Obesity, and Maternal Iron Supplements During Pregnancy eTable 9. Mutually Adjusted Associations Between Three Maternal Psychosocial Factors During Pregnancy and Neonatal Line Widths, Measured at the Cuspal, Mid-Crown, and Enamel-Dentine Junction (EDJ) Adjacent Portions eFigure 1. Correlations Between Measures of Maternal Psychosocial Factors eFigure 2. Distributions of Neonatal Line Width Measures eFigure 3. Correlations Between Measures of Neonatal Line Width eFigure 4. Tooth Position and the Neonatal Line Width Across Individuals eFigure 5. A Priori Power Analysis eFigure 6. Comparison of Associations Between Maternal Psychosocial Factors During Pregnancy and Neonatal Li [file jamanetwopen-e2129129-s001.pdf]

Supplemental Online Content

Mountain RV, Zhu Y, Pickett OR, et al. Association of maternal stress and social support during pregnancy with growth marks in children's primary tooth enamel. *JAMA Netw Open*. 2021;4(11):e2129129. doi:10.1001/jamanetworkopen.2021.29129

eMethods. Supplemental Methods
eResults. Supplemental Results
eTable 1. Perinatal Exposures Previously Investigated in Association With Neonatal Line Width: Results From 11 Studies
eTable 2. Characteristics of the Analytic Sample (n=70) and Comparison to the ALSPAC Full Sample (n=14 901)
eTable 3. Unadjusted Associations Between Perinatal Maternal Psychosocial Factors and Average Neonatal Line Widths Measured at the Cuspal, Middle, and Enamel-Dentine Junction (EDJ) Sections
eTable 4. Unadjusted Associations Between Previously Examined Perinatal Factors and Neonatal Line Widths at the Cuspal Portion
eTable 5. Unadjusted Associations Between Previously Examined Perinatal Factors and Neonatal Line Widths at the Middle Portion
eTable 6. Unadjusted Associations Between Previously Examined Perinatal Factors and Neonatal Line Widths at the Enamel-Dentine Junction (EDJ) Portion
eTable 7. Adjusted Associations Between Maternal Psychosocial Factors During Pregnancy and Neonatal Line Widths, Measured at the Cuspal, Mid-Crown, and Enamel-Dentine Junction (EDJ) Adjacent Portions, Controlling for Maternal Polygenic Risk Score (PRS) for Major Depressive Disorder
eTable 8. Associations Between Maternal Psychosocial Factors and Three Covariates Included in the Current Study: Gestational Age, Maternal Prepregnancy Obesity, and Maternal Iron Supplements During Pregnancy
eTable 9. Mutually Adjusted Associations Between Three Maternal Psychosocial Factors During Pregnancy and Neonatal Line Widths, Measured at the Cuspal, Mid-Crown, and Enamel-Dentine Junction (EDJ) Adjacent Portions
eFigure 1. Correlations Between Measures of Maternal Psychosocial Factors
eFigure 2. Distributions of Neonatal Line Width Measures
eFigure 3. Correlations Between Measures of Neonatal Line Width
eFigure 4. Tooth Position and the Neonatal Line Width Across Individuals
eFigure 5. A Priori Power Analysis
eFigure 6. Comparison of Associations Between Maternal Psychosocial Factors During Pregnancy and Neonatal Line Widths Before and After Controlling for Maternal Polygenic Risk Score (PRS) for Major Depressive Disorder
eReferences.

© 2021 Mountain RV et al. *JAMA Network Open.*

This supplemental material has been provided by the authors to give readers additional information about their work.

© 2021 Mountain RV et al. *JAMA Network Open*.

eMethods

Sample and Procedures

The Avon Longitudinal Study of Parents and Children (ALSPAC) consisted of pregnant women with expected delivery dates between April 1st, 1991 and December 31st, 1992 for the study. Recruitment occurred in both clinical and community settings[1]. Of the 14 541 pregnancies enrolled initially, 13 988 children were alive at 1 year of age. Subsequent enrollment phases were carried out since 1998 in an effort to extend the study to mother-child pairs meeting the inclusion criteria, but who did not join the study originally. The total ALSPAC sample consists of 14 901 children who were alive at 1 year of age.

Naturally shed primary teeth were collected from 4168 children at 5 and 7 years of age[2] as a part of the ALSPAC biobank. The large majority of these teeth were incisors, which are smaller in size and thus somewhat harder to analyze. In the current study, 81 deciduous canine teeth obtained from 71 children were analyzed in the laboratory in 2016. One outlier was subsequently excluded from any statistical analysis, as the neonatal line (NNL) measurements at all locations were outside 1.5 times the respective interquartile range. Consistent with the approach taken by Hassett et al[3], one tooth was selected randomly if the child contributed multiple teeth. The analytic sample for all primary analyses consisted of 70 teeth from 70 unique children.

Measures

Stressful life events. Exposures to partner emotional cruelty or loss of friend or a family member (defined as a child or partner) during pregnancy, both of which are key stressful life events conferring risks for adverse health outcomes, [4-6] were measured using five items taken from a 42-item stressful life events checklist. Participants were coded as having experienced partner emotional cruelty during pregnancy if they responded "yes" to the item "your partner was emotionally cruel to you" at either of the two time points of assessment. Exposure to loss of friend or family was determined through participants' affirmative responses to any of the three items at either time point: (1) your partner died; (2) one of your children died; (3) a friend or relative died. The report at 18 weeks' gestation focused on the mother's experience since she became pregnant, while the 8-week postnatal assessment covered exposures between mid-pregnancy and birth retrospectively. Of note, we included the items above to capture maternal exposure to interpersonal psychosocial stress and to complement our focus on social support. The included items were also the most commonly endorsed by participants in our analytic sample.

Psychopathology. At 12 weeks' gestation, mothers self-reported their lifetime history of 24 medical and psychiatric conditions by indicating whether or not they had the problem in the past. For our analyses, we included the following two items: maternal exposure to severe depression (yes/no) and any psychiatric problems, which was defined as being exposed to drug addiction, alcoholism, schizophrenia, anorexia nervosa, severe depression, or other psychiatric problem. Due to the low prevalence of all conditions except severe depression in the sample, we were unable to assess the associations between other mental disorders and tooth-based markers separately. Moreover, to assess levels of maternal depression or anxiety during pregnancy, we used data from the following well-established self-report measures of psychopathology symptoms: the Crown-Crisp Experiential Index (CCEI), which includes separate subscales for anxiety and depression[7,8]; and the Edinburgh Postnatal Depression Scale (EPDS)[9]. Consistent with criteria established in prior studies[8,10,11], children were coded as exposed to high maternal psychopathology symptoms if any of the following conditions was met: 1) the mother had a CCEI depression score greater than 9; 2) mother had a CCEI anxiety score greater than 10; or 3) mother had an EPDS score greater than 12.

Neighborhood disadvantage. Mothers reported levels of neighborhood quality at 8 weeks' gestation by indicating on a 4-item scale the degree to which they were concerned about the following in their neighborhood: burglary, mugging or robbery, sexual assault or pestering, and vandalism. Each item was coded on a 3-point scale (0=not worried, 1=fairly worried, 2=very worried) and scores were summed to create a total score. Additionally, mothers also reported on a 6-item scale how often they would agree with the following assessments about their impression of the neighborhood: lively, friendly, noisy, clean, attractive, and dirty/polluted, ranging from "usually" to "not at all." The negative items (noisy and dirty/polluted) were reverse coded such that higher scores reflected more disadvantage. We combined the two measures to capture levels of safety and quality of the environment. A sum score of general levels of neighborhood impoverishment was derived. Children were coded as exposed if the sum score was above 9, which corresponded to the 90th percentile in the total ALSPAC sample.

© 2021 Mountain RV et al. *JAMA Network Open.*

Social support. At 12 weeks' gestational and 8 weeks postpartum, mothers completed a 10-item questionnaire measuring their perceived levels of social support during pregnancy and shortly after birth. Several domains of social support, such as financial, emotional, and instrumental, were assessed. Sample items included: "My partner provides the emotional support I need," "I believe in moments of difficulty my neighbors would help me", and "If I was in financial difficulty, I know my family would help if they could." The scale also covered various sources of support, including partner, neighbors, and family. Mothers provided responses on a 4-point scale (from 1=exactly feel to 4=never feel). A total social support score was derived by summing across these items, with higher scores corresponding to a stronger sense of support. More details of the social support measure are provided elsewhere[12].

Covariate selection based on previous studied factors associated with NNL width
The NNL is one of the earliest and most prominent "stress lines" found in teeth. Previous research in anthropology and forensics has found that the NNL varies in width based on certain perinatal factors[13-17] (see **eTable 1**). Tooth type, age at time of death[16], sex[14], duration of gestation[3,13,17], seasonality of birth[3,14], mode of delivery[15,18], and maternal factors such as vitamin D levels[3], medications[14], and conditions including hypertension[3] and diabetes[19,20] have all been found to be associated with the width of the NNL. Notably, many of these studies have found significant associations[13-17] between a wider NNL and more "stressful" perinatal conditions, such as a complicated delivery, longer duration of delivery, pre-term births, and younger age at death. These findings suggest that characteristics of the NNL, already established as markers of stressful gestational events from anthropological research, could provide insight on perinatal disruptions that predict later psychopathology risk in pediatric populations.

In the current study, we considered a wide range of previously studied perinatal physiological factors as potential covariates. Because of the rich phenotype data collected by the ALSPAC, we were able to explore the associations between most factors identified from prior studies (eTable 1) and NNL width. Specifically, we tested 12 maternal exposures, four delivery related exposures, and 13 child exposures. We were unable to assess the following exposures due to data unavailability (i.e., the following previously examined factors from the literature were either not collected in ALSPAC or were not available to us at the time we completed the data analysis): use of antispasmodics during pregnancy, maternal age at pregnancy, number of children in family, past miscarriage, use of pregnancy sustaining medication, child blood transfusions, and child hypocalcemia. Further, the following perinatal factors were excluded because the cell size of being exposed was under 5: maternal diabetes, glycosuria, maternal distress during labor, vomiting during pregnancy, and child feeding problems. Additionally, we did not include maternal antidepressant use as a covariate, because none of the mothers in our sample reported use of psychopharmacological treatment. Considering that the number of preterm births in our analytic sample was low (n=3), we modeled gestational age as a continuous variable (unit: week). The assumption about a linear relationship between gestational age and NNL width was consistent with prior studies[21,22]. Overall, we made an effort to consider all available perinatal factors previously investigated to be related to NNL widths and thus possibly confounding the effects of maternal factors in our main analyses.

To study the NNL width as a biomarker for psychosocial exposures in psychiatry and pediatrics, we selected covariates by examining a comprehensive list of previously examined perinatal factors capturing maternal exposures, delivery related factors, and child exposures. Specifically, we estimated the unadjusted associations between each of the factors and NNL widths at the cuspal (**eTable 4**), middle (**eTable 5**), and EDJ portions (**eTable 6**). Out of many tested associations, only the following three factors showed a significant association with NNL widths at the middle portion at $p<0.05$: maternal iron supplements during pregnancy (yes/no, $\beta = 2.28$, p=0.01), maternal prepregnancy obesity (yes/no, $\beta = -3.91$, p=0.048), and gestational age (weeks, $\beta = -0.56$, p=0.02) (**eTables 4-6**).

Associations between the three selected covariates and the maternal psychosocial factors we considered are shown in **eTable 8**. Mothers who reported taking iron supplements during pregnancy were more likely to be exposed to some maternal psychosocial stressors, especially lifetime psychiatric problems ($\chi 2$ test p=0.04). However, caution is required when interpreting these results, due to very small cell counts.

Genotyping procedure and quality control
Blood samples were collected from ALSPAC mothers during routine antenatal care and subsequent clinic visits. DNA was extracted via a phenol–chloroform, salting- out or guanidine hydrocholoride extraction methods[23].

© 2021 Mountain RV et al. *JAMA Network Open.*

Approximately 10000 ALSPAC mothers were genotyped using the Illumina human660W-quad array at Centre National de Génotypage (CNG). Genotypes were called with Illumina GenomeStudio. Initially, quality control measures were carried out on 10,015 participants and 557,124 directly genotyped SNPs using PLINK (v1.07). Quality control steps included: removing SNPs with >5% missingness, Hardy-Weinberg equilibrium *p*-value <1.0e-06, or minor allele frequency (MAF) <1%; excluding samples with >5% missingness, indeterminate X chromosome heterozygosity, or extreme autosomal heterozygosity. After quality control filters, 9,048 participants and 526,688 SNPs were retained for subsequent phasing and imputation. Imputation was performed using Impute V2.2.2, with 1000 genomes (Phase 1, version 3) as the reference panel, which included 2,186 reference haplotypes[24,25].

Polygenic risk score (PRS) generation and selection

To construct PRS for major depressive disorder in mothers, we used the most recent summary statistics from the Psychiatric Genomics Consortium GWAS of major depressive disorder (MDD), wave 2 (PGC-MDD2; 43,204 cases; 95,680 controls), and UK Biobank (127,552 cases; 233,763 controls) [26,27]. Additional genomic quality control steps preceding score construction included: removing imputed SNPs with low imputation quality (metric score <0.8), and any SNP with MAF <1%, call rate <95%, or HWE p<1e-6. Prior to calculating the PRS, we pruned the SNPs based on linkage disequilibrium patterns using p-value informed clump-based method in PLINK v1.90 (with r^2 threshold set to 0.25 within a 250kb window). To create the PRS, we summed the number of risk alleles (0, 1, or 2) for each SNP at a given p-value threshold, weighted by the log odds ratio reported for MDD in the GWAS by PGC[27]. In the ALSPAC cohort, we selected independent subsets of SNPs from GWAS summary data at a significance threshold of p_T <0.001, which explains the most phenotypic variance in the discovery sample, meaning the GWAS performed by Wray et al as shown in another more recent GWAS of depression[28]. All PRS were standardized prior to analysis; thus, PRS values can be interpretated on the scale of each one-unit standard deviation difference in the genetic score of ALSPAC mothers.

Sensitivity analysis

We performed two sets of sensitivity analyses. Because maternal genetic liability for depression could be linked to both maternal psychopathology and potentially tooth-based markers, leading to a spurious association between maternal psychosocial stress and NNL widths, we performed an analysis controlling for maternal polygenic risk scores (PRS) for depression in the subsample of children with maternal genotype data available (n=54). PRS values were derived, as described above. Results from these analyses are shown in **eTable 7 and eFigure 6.**

To further examine the relative contributions of each maternal psychosocial factor on NNL widths, we additionally performed a mutually adjusted regression analysis. By mutually adjusted regression, we mean an analysis where multiple exposures were included in the same regression model. In the mutually adjusted model, we included the following three psychosocial factors simultaneously, in addition to the covariates: maternal severe lifetime depression history, maternal depression or anxiety at gestational 32 weeks, and high social support at 8 weeks postpartum. Of note, because any lifetime psychiatric problem and severe lifetime depression history were highly correlated, we only included maternal severe lifetime depression history. The remaining three variables were not highly collinear, as indicated by variance inflation factors (VIFs) under 2 in the mutually adjusted models ($VIF_{depression}$=1.19, $VIF_{anxiety\ or\ depression}$=1.11, $VIF_{support}$=1.05). Results from these analyses are shown in **eTable 9**.

© 2021 Mountain RV et al. *JAMA Network Open.*

eResults

Correlations between maternal psychosocial factors
Psychosocial factors were weakly to strongly correlated, ranging from 0.02 to 0.81(**eFigure 1**). The maternal psychopathology measures clustered together, with the highest correlation occurring between the reports of severe lifetime depression history and any psychiatric problems ($r_{tetrachoric}$ = 0.82). In other words, measures of "trait" (or history of) psychopathology before pregnancy were correlated with the "state" (or symptoms of) psychopathology during pregnancy.

The lack of correlation between types of psychosocial factors indicated that they likely captured separate domains of experiences during pregnancy, warranting their inclusion in the analyses. Social support, as a protective factor, was generally negatively associated with psychosocial stress, as expected.

Characteristics of tooth-based markers
Deciduous canines came from each of the four dental quadrants ($n_{upperright}$=18, $n_{upperleft}$ = 13, $n_{lowerleft}$ = 22, $n_{lowerright}$=17). Consistent with the findings of Hassett and colleagues[3], NNL widths varied by location along the tooth crown (i.e., cuspal, middle, and the EDJ portions) (**eFigure 2**). Measures were correlated within each portion, but distinct across portions, suggesting that tooth-based markers at the three portions may capture different biological signatures (**eFigure 3**). Across participants, tooth quadrant position was unassociated with any NNL measure (F_{cusp}=0.95, p_{cusp}=0.42; F_{mid}=0.45, p_{mid}=0.72; F_{EDJ}=0.15, p_{EDJ}=0.93; **eFigure 4**).

Power analysis
Before performing any empirical analysis, we assessed the statistical power *a priori*, by considering a range of R^2 values and varying sample sizes. With the sample size of the current study (n=70), our study would be sufficiently powered (i.e., with power above 80%) to detect an effect of R^2 = 0.1 or larger in a simple linear regression model (**eFigure 5**). To detect smaller effects, such as R^2 < 0.05, a sample size of 200 or higher would be required.

Sensitivity analysis
Results from the mutually adjusted model suggest that severe lifetime depression history and high social support postpartum were still associated with NNL width at the EDJ portion (**eTable 9**). Specifically, the positive effect estimate associated with severe lifetime depression history was twice as large as the negative effect estimate corresponding to having higher social support after birth ($\beta_{depression}$=3.18, $p_{depression}$=0.001, 95% CI [1.30, 5.07]; $\beta_{support}$=-1.53, $p_{support}$=0.043, 95% CI [-3.02, -0.05]). Maternal depression or anxiety at 32 gestational weeks was no longer significantly associated with NNL widths.

© 2021 Mountain RV et al. *JAMA Network Open.*

eTable 1. Perinatal exposures previously investigated in association with neonatal line width: Results from 11 studies.

	Exposure	Effect on NNL Width	Reference
Maternal Exposures	Obesity	*Negative*	Hassett et al. 2020
	Use of antispasmodics during pregnancy	*Negative*	Kurek et al. 2015
	Vitamin D levels	*Negative*	Hassett et al. 2020
	Alcohol consumption during pregnancy	*Positive*	Behie & Miszkiewicz 2019
	Diabetes	*Positive*	Noren 1984; Noren et al. 1978
	History of hypertension	*Positive*	Hassett et al. 2020
	Hypertension during pregnancy	*Positive*	Hassett et al. 2020
	Age at delivery	*No effect*	Behie & Miszkiewicz 2019; Hassett et al. 2020; Kurek et al. 2015
	Age at pregnancy	*No effect*	Behie & Miszkiewicz 2019
	BMI	*No effect*	Hassett et al. 2020
	Glycosuria	*No effect*	Hassett et al. 2020
	Height	*No effect*	Behie & Miszkiewicz 2019; Hassett et al. 2020
	Illness/infection during pregnancy	*No effect*	Behie & Miszkiewicz 2019; Hassett et al. 2020; Kurek et al. 2015
	Injury or shock during pregnancy	*No effect*	Hassett et al. 2020
	Iron supplements during pregnancy	*No effect*	Kurek et al. 2015
	Number of children in family	*No effect*	Kurek et al. 2015
	Past miscarriage	*No effect*	Behie et al. 2019
	Stressful event during pregnancy	*No effect*	Behie et al. 2019
	Use of pregnancy sustaining medication	*No effect*	Kurek et al. 2015
	Vitamin supplements during pregnancy	*No effect*	Kurek et al. 2015
	Vomiting during pregnancy	*No effect*	Hassett et al. 2020
	Weight	*No effect*	Hassett et al. 2020

© 2021 Mountain RV et al. *JAMA Network Open.*

	Exposure	Effect on NNL Width	Reference
Delivery Exposures	Cesarean delivery	*Negative*	Eli et al. 1989; Canturk et al. 2014
		No effect	Behie & Miszkiewicz 2019; Hassett et al. 2020; Hurnanen et al. 2017; Kurek et al. 2015; Zanolli et al. 2011
	Duration of delivery	*Negative*	Hurnanen et al. 2017
		No effect	Behie & Miszkiewicz 2019; Hassett et al. 2020
	Operative delivery	*Positive*	Eli et al. 1989
		No effect	Behie & Miszkiewicz 2019; Hassett et al. 2020; Hurnanen et al. 2017; Kurek et al. 2015; Zanolli et al. 2011
	Induction	*No effect*	Behie & Miszkiewicz 2019
	Maternal distress during labor	*No effect*	Hassett et al. 2020
Child Exposures	Autumn birth	*Negative*	Hassett et al. 2020
	Post-term birth	*Negative*	Hassett et al. 2020
		No effect	Behie & Miszkiewicz 2019; Zanolli et al. 2011
	Resuscitated	*Negative*	Hassett et al. 2020
	Female sex	*Positive*	Kurek et al. 2015
		No effect	Behie & Miszkiewicz 2019; Hassett et al. 2020; Hurnanen et al. 2017; Zanolli et al. 2011
	Low birth weight	*Positive*	Norén 1983
		No effect	Hassett et al. 2020
	Preterm birth (before 37th week)	*Positive*	Hassett et al. 2020; Zanolli et al. 2011
		No effect	Behie & Miszkiewicz 2019
	Winter birth	*Positive*	Hassett et al. 2020; Kurek et al. 2015
	Abnormal fetal heart rate	*No effect*	Hassett et al. 2020
	APGAR score	*No effect*	Hassett et al. 2020
	Birth order	*No effect*	Behie & Miszkiewicz 2019; Hassett et al. 2020; Kurek et al. 2015
	Blood transfusions	*No effect*	Ranggård et al. 1995
	Feeding problems	*No effect*	Hassett et al. 2020
	Hypocalcemia	*No effect*	Hassett et al. 2020; Ranggård et al. 1995
	Jaundice	*No effect*	Hassett et al. 2020
	Length/height	*No effect*	Behie & Miszkiewicz 2019; Hassett et al. 2020
	Pyrexia	*No effect*	Hassett et al. 2020

© 2021 Mountain RV et al. *JAMA Network Open.*

eTable 2. Characteristics of the analytic sample (n=70) and comparison to the ALSPAC full sample (n=14 901).

	**Analytic sample** N=70 **N (%)**	**ALSPAC** N=14 901 **N (%)**	***p*-value**
Sociodemographic characteristics			
Sex			1
Males	36 (51.4)	7542 (51.3)	
Females	34 (48.6)	7152 (48.7)	
Race			0.948
Non-white	4 (6.0)	611 (5.1)	
White	63 (94.0)	11488 (94.9)	
Home Ownership			0.002
Mortgage/own home	61 (92.4)	9885 (73.2)	
Rent home	5 (7.6)	3144 (23.3)	
Other	0 (0.0)	472 (3.5)	
Maternal Education			<0.001
less than O-level	6 (8.8)	3735 (30.0)	
O-level	21 (30.9)	4303 (34.6)	
A-level	19 (27.9)	2795 (22.5)	
Degree or Above	22 (32.4)	1603 (12.9)	
Pregnancy and birth related factors			
Gestational age			0.129
Early term	3 (4.4)	1440 (9.9)	
Full term	57 (83.8)	12073 (83.0)	
Post term	8 (11.8)	1038 (7.1)	
Birth order			0.783
first child	27 (42.2)	5800 (44.7)	
second or later	37 (57.8)	7182 (55.3)	
Maternal age at birth			0.064
Ages 15-19	0 (0.0)	650 (4.7)	
Ages 20-35	60 (88.2)	12303 (88.4)	
Ages 36+	8 (11.8)	960 (6.9)	
Alcohol consumption during pregnancy			0.094
Never	16 (23.5)	4692 (33.0)	
Occasional or often	36 (52.9)	5753 (40.4)	
Rarely	16 (23.5)	3779 (26.6)	
Birth weight (g)			0.59
< 3000	20 (28.6)	3649 (24.8)	
3000 - 3499	24 (34.3)	4924 (33.5)	
3500 - 3999	16 (22.9)	4382 (29.8)	
>= 4000	10 (14.3)	1735 (11.8)	

© 2021 Mountain RV et al. *JAMA Network Open.*

	Analytic sample<br>N=70<br>N (%)	ALSPAC<br>N=14901<br>N (%)	*p*-value
Maternal physiological factors			
Iron supplement during pregnancy			0.949
No	33 (48.5)	6148 (49.7)	
Yes	35 (51.5)	6232 (50.3)	
Obesity before pregnancy			1
No	63 (94.0)	11034 (94.4)	
Yes	4 (6.0)	649 (5.6)	
Maternal psychosocial factors during pregnancy			
Partner emotional cruelty			0.712
Unexposed	51 (86.4)	9466 (88.8)	
Exposed	8 (13.6)	1194 (11.2)	
Loss of friend or family			0.526
Unexposed	50 (74.6)	10524 (78.5)	
Exposed	17 (25.4)	2875 (21.5)	
Severe lifetime depression history			0.875
Unexposed	61 (89.7)	11307 (91.0)	
Exposed	7 (10.3)	1119 (9.0)	
Any lifetime psychiatric problem			0.903
Unexposed	59 (86.8)	10933 (88.0)	
Exposed	9 (13.2)	1493 (12.0)	
Maternal depression or anxiety (18 gestational weeks)			0.181
Unexposed	49 (89.1)	9493 (81.1)	
Exposed	6 (10.9)	2206 (18.9)	
Maternal depression or anxiety (32 gestational weeks)			0.069
Unexposed	58 (89.2)	9233 (79.3)	
Exposed	7 (10.8)	2403 (20.7)	
Neighborhood disadvantage			0.596
Unexposed	57 (93.4)	11432 (90.7)	
Exposed	4 (6.6)	1179 (9.3)	
High social support (12 gestational weeks)			
High support	3 (4.8)	1304 (11.3)	0.159
Other	59 (95.2)	10207 (88.7)	
High social support (8 weeks postpartum)			0.428
High support	9 (14.5)	1147 (10.6)	
Other	53 (85.5)	9680 (89.4)	

Note. p-values were obtained from χ^2 tests comparing the distribution of covariates in the analytic sample (n=70) versus that in the rest of the ALSPAC sample. Time periods for maternal psychosocial factors during pregnancy are also indicated when these measures were collected at multiple time points.

eTable 3. Unadjusted associations between perinatal maternal psychosocial factors and average neonatal line widths measured at the cuspal, middle, and enamel-dentine junction (EDJ) sections.

Exposure	Beta	P-value	95% CI lower bound	95% CI upper bound	R^2
Cuspal portion (Mean = 11.83, SD =4.97)					
Partner emotional cruelty	0.68	0.7301	-3.24	4.60	<0.01
Loss of a family member or friend	0.96	0.4958	-1.84	3.76	0.01
Severe lifetime depression history	1.77	0.3751	-2.19	5.73	0.01
Any lifetime psychiatric problem	1.76	0.3237	-1.78	5.31	0.01
Maternal depression or anxiety 18 gestational weeks	0.61	0.7763	-3.69	4.92	<0.01
Maternal depression or anxiety 32 gestational weeks	2.73	0.1698	-1.20	6.65	0.03
Neighborhood disadvantage	-0.12	0.9651	-5.47	5.23	<0.01
Social support 12 gestational weeks	-3.71	0.1681	-9.03	1.61	0.03
Social support 8 weeks postpartum	-2.82	0.1139	-6.35	0.70	0.04
Middle portion (Mean = 9.87, SD =3.8)					
Partner emotional cruelty	0.56	0.7142	-2.47	3.58	<0.01
Loss of a family member or friend	-0.24	0.8265	-2.41	1.93	<0.01
Severe lifetime depression history	2.40	0.1175	-0.62	5.42	0.04
Any lifetime psychiatric problem	2.33	0.0899	-0.37	5.02	0.04
Maternal depression or anxiety 18 gestational weeks	0.00	0.9978	-3.33	3.32	<0.01
Maternal depression or anxiety 32 gestational weeks	2.59	0.0908	-0.42	5.60	0.04
Neighborhood disadvantage	2.08	0.3163	-2.04	6.21	0.02
Social support 12 gestational weeks	-2.88	0.2037	-7.37	1.61	0.03
Social support 8 weeks postpartum	-1.68	0.2418	-4.52	1.16	0.02
EDJ portion (Mean = 7.34, SD =2.46)					
Partner emotional cruelty	0.17	0.8548	-1.73	2.08	<0.01
Loss of a family member or friend	0.09	0.8971	-1.31	1.49	<0.01
**Severe lifetime depression history**	**3.31**	**0.0005**	**1.50**	**5.12**	**0.17**
**Any lifetime psychiatric problem**	**2.57**	**0.0030**	**0.91**	**4.24**	**0.13**
Maternal depression or anxiety 18 gestational weeks	1.54	0.1468	-0.56	3.64	0.03
**Maternal depression or anxiety 32 gestational weeks**	**2.62**	**0.0070**	**0.74**	**4.50**	**0.11**
Neighborhood disadvantage	1.18	0.3777	-1.48	3.84	0.01
Social support 12 gestational weeks	-1.07	0.4427	-3.84	1.70	0.01
**Social support 8 weeks postpartum**	**-1.80**	**0.0363**	**-3.49**	**-0.12**	**0.07**

© 2021 Mountain RV et al. *JAMA Network Open.*

eTable 4. Unadjusted associations between previously examined perinatal factors and neonatal line widths at the cuspal portion.

	Exposure	Coding in sample	Beta	SE	95% CI lower bound	95% CI upper bound	F statistic	P-value	R-squared
**Maternal Exposures**	Alcohol consumption during pregnancy	Occasional or often / Rarely or never	-0.05	1.21	-2.47	2.37	0.00	0.97	0.000
	Hypertension	History of hypertension or hypertension during pregnancy, yes/no	0.48	1.53	-2.57	3.53	0.10	0.75	0.002
	Maternal vitamin D levels	Vitamin D total in pregnancy, nmol/l	-0.02	0.03	-0.08	0.05	0.25	0.62	0.007
	Injury/shock during pregnancy	Yes/No	-0.06	1.58	-3.22	3.10	0.00	0.97	0.000
	Maternal age at delivery	Continuous, year	-0.10	0.17	-0.43	0.23	0.36	0.55	0.005
	Maternal BMI	Prepregnancy continuous BMI	-0.17	0.17	-0.51	0.17	1.00	0.32	0.015
	Maternal height	Continuous, cm	0.06	0.09	-0.13	0.25	0.42	0.52	0.006
	Maternal illness/infection during pregnancy	Yes/No	0.55	1.24	-1.93	3.03	0.20	0.66	0.003
	Maternal iron supplements	Yes/No	2.06	1.19	-0.31	4.42	3.01	0.09	0.044
	Maternal obesity	Prepregnancy BMI > 31, yes/no	-4.94	2.51	-9.95	0.06	3.89	0.05	0.056
	Maternal vitamin supplements	Yes/No	-1.06	1.39	-3.83	1.71	0.58	0.45	0.009
	Maternal weight	Prepregnancy continuous weight, kg	-0.03	0.06	-0.14	0.09	0.21	0.65	0.003
**Delivery Exposures**	Operative delivery	Yes/No	0.28	1.57	-2.93	3.49	0.03	0.86	0.001
	Cesarean delivery	Yes/No	2.19	1.97	-1.83	6.22	1.24	0.27	0.040
	Duration of delivery	Duration of first and second stage labor combined, hour	-0.15	0.13	-0.42	0.12	1.24	0.28	0.047
	Induction	Yes/No	2.28	2.12	-2.05	6.61	1.15	0.29	0.037

© 2021 Mountain RV et al. *JAMA Network Open.*

	Exposure	Coding in sample	Beta	SE	95% CI lower	95% CI upper	F statistic	P-value	R-squared
**Child Exposures**	Female sex of child	Yes/No	-0.47	1.20	-2.86	1.91	0.16	0.69	0.002
	Birthweight	Continuous, g	0.00	0.00	0.00	0.00	0.04	0.84	0.001
	Gestational age	Continuous, week	-0.37	0.32	-1.01	0.27	1.36	0.25	0.020
	Winter birth	Yes/No	0.39	1.79	-3.17	3.96	0.05	0.83	0.001
	Autumn birth	Yes/No	-1.63	1.70	-5.02	1.76	0.92	0.34	0.013
	Abnormal fetal heart rate	Yes/No	0.54	1.69	-2.94	4.02	0.10	0.75	0.004
	APGAR score (1 minute)	Continuous score	-0.07	0.53	-1.15	1.01	0.02	0.89	0.001
	APGAR score (5 minute)	Continuous score	0.38	1.11	-1.88	2.65	0.12	0.73	0.004
	Birth order	Ordinal, 1-4	0.09	0.78	-1.48	1.65	0.01	0.91	0.000
	Child length/height	Continuous length, cm	-0.49	0.38	-1.26	0.28	1.65	0.21	0.044
	Child resuscitated	Yes/No	-1.90	1.66	-5.28	1.49	1.31	0.26	0.042
	Jaundice	Yes/No	0.31	1.69	-3.15	3.77	0.03	0.86	0.001
	Prexia	Yes/No	-0.04	1.62	-3.36	3.27	0.00	0.98	0.000

© 2021 Mountain RV et al. *JAMA Network Open.*

eTable 5. Unadjusted associations between previously examined perinatal factors and neonatal line widths at the middle portion.

	Exposure	Coding in sample	Beta	SE	95% CI lower bound	95% CI upper bound	F statistic	P-value	R-squared
**Maternal Exposures**	Alcohol consumption during pregnancy	Occasional or often / Rarely or never	-0.49	0.94	-2.36	1.38	0.27	0.60	0.004
	Hypertension	History of hypertension or hypertension during pregnancy, yes/no	-0.91	1.17	-3.25	1.43	0.60	0.44	0.009
	Maternal vitamin D levels	Vitamin D total in pregnancy, nmol/l	-0.02	0.03	-0.08	0.04	0.54	0.47	0.015
	Injury or shock during pregnancy	Yes/No	1.65	1.21	-0.77	4.07	1.86	0.18	0.030
	Maternal age at delivery	Continuous, year	-0.08	0.13	-0.34	0.17	0.42	0.52	0.006
	Maternal BMI	Prepregnancy continuous BMI	-0.16	0.13	-0.42	0.10	1.50	0.22	0.023
	Maternal height	Continuous, cm	0.11	0.07	-0.04	0.25	2.21	0.14	0.032
	Maternal illness/infection during pregnancy	Yes/No	-0.39	0.98	-2.33	1.56	0.16	0.69	0.002
	Maternal iron supplements	Yes/No	2.28	0.89	0.50	4.07	6.54	0.01	0.090
	Maternal obesity	Prepregnancy BMI > 31, yes/no	-3.91	1.94	-7.78	-0.03	4.06	0.05	0.059
	Maternal vitamin supplements	Yes/No	-0.03	1.07	-2.18	2.12	0.00	0.98	0.000
	Maternal weight	Prepregnancy continuous weight, kg	-0.01	0.04	-0.10	0.07	0.11	0.74	0.002
**Delivery Exposures**	Operative delivery	Yes/No	-0.78	1.26	-3.35	1.80	0.38	0.54	0.013
	Cesarean delivery	Yes/No	-0.19	1.62	-3.50	3.12	0.01	0.91	0.000
	Duration of delivery	Duration of first and second stage labor combined, hour	-0.19	0.11	-0.42	0.05	2.72	0.11	0.098
	Induction	Yes/No	0.16	1.74	-3.39	3.72	0.01	0.93	0.000

© 2021 Mountain RV et al. *JAMA Network Open.*

	Exposure	Coding in sample	Beta	SE	95% CI lower bound	95% CI upper bound	F statistic	P-value	R-squared
**Child Exposures**	Female sex of child	Yes/No	0.04	0.92	-1.78	1.87	0.00	0.96	0.000
	Birthweight	Continuous, g	0.00	0.00	0.00	0.00	0.01	0.92	0.000
	Gestational age	Continuous, week	-0.56	0.24	-1.04	-0.08	5.50	0.02	0.077
	Winter birth	Yes/No	1.33	1.36	-1.38	4.04	0.96	0.33	0.014
	Autumn birth	Yes/No	-2.05	1.28	-4.61	0.51	2.56	0.11	0.036
	Abnormal fetal heart rate	Yes/No	1.35	1.45	-1.62	4.33	0.88	0.36	0.033
	APGAR score (1 minute)	Continuous score	0.14	0.42	-0.72	1.01	0.11	0.74	0.004
	APGAR score (5 minute)	Continuous score	0.64	0.89	-1.17	2.45	0.52	0.48	0.017
	Birth order	Ordinal, 1-4	-0.14	0.61	-1.36	1.07	0.06	0.81	0.001
	Child length/height	Continuous length, cm	-0.34	0.30	-0.95	0.26	1.31	0.26	0.035
	Child resuscitated	Yes/No	-1.91	1.32	-4.60	0.79	2.09	0.16	0.065
	Jaundice	Yes/No	0.87	1.36	-1.89	3.64	0.42	0.52	0.014
	Prexia	Yes/No	1.78	1.27	-0.81	4.36	1.97	0.17	0.062

© 2021 Mountain RV et al. *JAMA Network Open.*

eTable 6. Unadjusted associations between previously examined perinatal factors and neonatal line widths at the enamel-dentine junction (EDJ) portion.

	Exposure	Coding in sample	Beta	SE	95% CI lower bound	95% CI upper bound	F statistic	P-value	R-squared
**Maternal Exposures**	Alcohol consumption during pregnancy	Occasional or often / Rarely or never	-0.36	0.60	-1.56	0.85	0.35	0.56	0.005
	Hypertension	History of hypertension or hypertension during pregnancy, yes/no	-0.17	0.75	-1.68	1.33	0.05	0.82	0.001
	Maternal vitamin D levels	Vitamin D total in pregnancy, nmol/l	0.01	0.02	-0.02	0.04	0.45	0.51	0.012
	Injury or shock during pregnancy	Yes/No	-0.10	0.79	-1.68	1.48	0.01	0.90	0.000
	Maternal age at delivery	Continuous, year	-0.10	0.08	-0.26	0.07	1.44	0.24	0.021
	Maternal BMI	Prepregnancy continuous BMI	-0.08	0.09	-0.25	0.09	0.91	0.34	0.014
	Maternal height	Continuous, cm	0.04	0.05	-0.06	0.13	0.59	0.44	0.009
	Maternal illness/infection during pregnancy	Yes/No	0.25	0.62	-0.99	1.50	0.17	0.68	0.003
	Maternal iron supplements	Yes/No	0.91	0.59	-0.28	2.10	2.34	0.13	0.034
	Maternal obesity	Prepregnancy BMI > 31, yes/no	-1.52	1.28	-4.08	1.03	1.42	0.24	0.021
	Maternal vitamin supplements	Yes/No	-0.13	0.69	-1.50	1.25	0.03	0.85	0.001
	Maternal weight	Prepregnancy continuous weight, kg	-0.01	0.03	-0.07	0.05	0.17	0.68	0.003
**Delivery Exposures**	Operative delivery	Yes/No	0.19	0.82	-1.48	1.87	0.06	0.82	0.002
	Cesarean delivery	Yes/No	0.54	1.05	-1.59	2.68	0.27	0.61	0.009
	Duration of delivery	Duration of first and second stage labor combined, hour	-0.05	0.07	-0.19	0.10	0.40	0.53	0.016
	Induction	Yes/No	-0.23	1.13	-2.53	2.07	0.04	0.84	0.001

© 2021 Mountain RV et al. *JAMA Network Open.*

	Exposure	Coding in sample	Beta	SE	95% CI lower bound	95% CI upper bound	F statistic	P-value	R-squared
**Child Exposures**	Female sex of child	Yes/No	-0.09	0.59	-1.27	1.09	0.02	0.88	0.000
	Birthweight	Continuous, g	0.00	0.00	0.00	0.00	0.22	0.64	0.003
	Gestational age	Continuous, week	-0.25	0.16	-0.57	0.07	2.46	0.12	0.036
	Winter birth	Yes/No	-0.17	0.88	-1.93	1.60	0.04	0.85	0.001
	Autumn birth	Yes/No	-0.76	0.84	-2.43	0.92	0.82	0.37	0.012
	Abnormal fetal heart rate	Yes/No	0.53	0.89	-1.30	2.35	0.35	0.56	0.013
	APGAR score (1 minute)	Continuous score	-0.06	0.28	-0.63	0.50	0.05	0.82	0.002
	APGAR score (5 minute)	Continuous score	0.31	0.58	-0.87	1.49	0.28	0.60	0.009
	Birth order	Ordinal, 1-4	-0.49	0.38	-1.25	0.27	1.64	0.20	0.026
	Child length/height	Continuous length, cm	-0.11	0.18	-0.49	0.26	0.38	0.54	0.010
	Child resuscitated	Yes/No	-1.57	0.84	-3.28	0.14	3.52	0.07	0.105
	Jaundice	Yes/No	0.39	0.88	-1.41	2.18	0.19	0.67	0.006
	Prexia	Yes/No	0.01	0.85	-1.72	1.74	0.00	0.99	0.000

© 2021 Mountain RV et al. *JAMA Network Open.*

eTable 7. Adjusted associations between maternal psychosocial factors during pregnancy and neonatal line widths, measured at the cuspal, mid-crown, and enamel-dentine junction (EDJ) adjacent portions, controlling for maternal polygenic risk score (PRS) for major depressive disorder.

Exposure	Beta	SE	P-value	FDR adjusted p-value	95% CI lower bound	95% CI upper bound	Model overall R^2
Cuspal portion (Mean = 11.83, SD =4.97)							
Severe lifetime depression history	2.64	2.50	0.2962	0.36	-2.39	7.66	0.13
Any lifetime psychiatric problem	2.58	2.14	0.2349	0.31	-1.73	6.88	0.14
Maternal depression or anxiety, 32 gestational weeks	3.01	2.26	0.1887	0.31	-1.53	7.55	0.14
**High social support after birth**	-6.03	2.17	**0.0080**	0.052	-10.41	-1.66	0.24
Middle portion (Mean = 9.87, SD =3.8)							
Severe lifetime depression history	1.25	1.82	0.4943	0.49	-2.41	4.91	0.25
Any lifetime psychiatric problem	1.88	1.55	0.2316	0.31	-1.24	4.99	0.26
Maternal depression or anxiety, 32 gestational weeks	1.32	1.65	0.4269	0.47	-2.00	4.65	0.25
High social support after birth	-3.15	1.64	0.0622	0.12	-6.47	0.17	0.35
EDJ portion (Mean = 7.34, SD =2.46)							
**Severe lifetime depression history**	2.72	1.17	**0.0239**	0.10	0.38	5.07	0.23
**Any lifetime psychiatric problem**	2.18	1.01	**0.0360**	0.11	0.15	4.22	0.22
**Maternal depression or anxiety, 32 gestational weeks**	2.19	1.08	**0.0477**	0.11	0.02	4.35	0.21
**High social support 8 weeks postpartum**	-2.84	1.03	**0.0087**	0.052	-4.93	-0.76	0.27

Note. Bolded values indicate associations significant at p<0.05. Mean and standard deviation of each NNL portion are noted in the section header to provide a reference for interpreting the magnitude of effect estimates. In addition to the baseline covariates included in the main analyses, we additionally controlled for maternal polygenic risk score for major depressive disorder in a subsample of children with maternal genotype available (n=54).

© 2021 Mountain RV et al. *JAMA Network Open.*

eTable 8. Associations between maternal psychosocial factors and three covariates included in the current study: gestational age, maternal prepregnancy obesity, and maternal iron supplements during pregnancy.

	**Gestational age**			**Maternal prepregnancy obesity**			**Maternal iron supplements during pregnancy**		
	Early or full term n=42	Late or post term n=26	χ2 p-value	BMI < 30 n=63	BMI >= 30 n=4	χ2 p-value	No n=33	Yes n=35	χ2 p-value
	Exposed n (%)			Exposed n (%)			Exposed n (%)		
Partner emotional cruelty	3 (8.3)	5 (21.7)	0.281	8 (14.8)	0 (0.0)	0.938	4 (13.8)	4 (13.3)	1
Loss of friend or family	13 (31.0)	4 (16.0)	0.285	17 (27.4)	0 (0.0)	0.532	8 (24.2)	9 (26.5)	1
Severe lifetime depression history	4 (9.5)	3 (11.5)	1	6 (9.5)	1 (25.0)	0.89	1 (3.0)	6 (17.1)	0.13
**Any lifetime psychiatric problem**	4 (9.5)	5 (19.2)	0.436	8 (12.7)	1 (25.0)	1	**1 (3.0)**	**8 (22.9)**	**0.04**
Maternal depression or anxiety (gestational 18 weeks)	4 (10.3)	2 (8.3)	1	6 (10.3)	0 (0.0)	1	1 (3.2)	5 (15.6)	0.212
Maternal depression or anxiety (gestational 32 weeks)	6 (14.3)	1 (3.8)	0.334	7 (11.1)	0 (0.0)	1	2 (6.1)	5 (14.3)	0.474
Neighborhood disadvantage	3 (8.1)	1 (4.2)	0.938	4 (7.1)	0 (0.0)	1	0 (0.0)	4 (12.5)	0.147
High social support (gestational 12 weeks)	2 (5.3)	1 (4.2)	1	2 (3.5)	0 (0.0)	1	3 (9.4)	0 (0.0)	0.26
High social support (8 weeks postpartum)	5 (13.9)	4 (15.4)	1	9 (15.8)	0 (0.0)	0.895	4 (13.3)	5 (15.6)	1

Note. Bolded values indicate associations significant at p<0.05. Although gestational age was modeled as a continuous variable (unit: weeks) in the regression analyses, we presented a dichotomous version of the variable to allow for easier comparison across the three covariates. Maternal prepregnancy obesity and iron supplements during pregnancy were both coded as dichotomous variables, indicating the presence versus absence of the two conditions.

© 2021 Mountain RV et al. *JAMA Network Open.*

eTable 9. Mutually adjusted associations between three maternal psychosocial factors during pregnancy and neonatal line widths, measured at the cuspal, mid-crown, and enamel-dentine junction (EDJ) adjacent portions

Variable	Beta	SE	P-value	95% CI lower bound	95% CI upper bound
Cuspal portion (Mean = 11.83, SD =4.97)					
Intercept	33.65	16.23	0.0429	1.12	66.19
Severe lifetime depression history	1.29	2.23	0.5652	-3.17	5.75
Maternal depression or anxiety, 32 gestational weeks	1.52	2.15	0.4837	-2.80	5.84
High social support, 8 weeks postpartum	-3.16	1.76	0.0782	-6.68	0.37
Gestational age (weeks)	-0.56	0.41	0.1754	-1.38	0.26
Maternal obesity before pregnancy	-4.03	2.67	0.1372	-9.39	1.33
Maternal iron supplements during pregnancy	1.18	1.32	0.3741	-1.46	3.82
Middle portion (Mean = 9.87, SD =3.8)					
Intercept	50.27	11.26	<0.0001	27.70	72.84
Severe lifetime depression history	2.41	1.54	0.1241	-0.68	5.51
Maternal depression or anxiety, 32 gestational weeks	1.66	1.49	0.2718	-1.34	4.65
High social support, 8 weeks postpartum	-1.91	1.22	0.1232	-4.35	0.54
Gestational age (weeks)	-1.04	0.28	0.0005	-1.61	-0.48
Maternal obesity before pregnancy	-2.18	1.85	0.2441	-5.90	1.53
Maternal iron supplements during pregnancy	2.16	0.91	0.0217	0.33	3.99
EDJ portion (Mean = 7.34, SD =2.46)					
Intercept	21.54	6.84	0.0027	7.82	35.26
**Severe lifetime depression history**	**3.18**	**0.94**	**0.0013**	**1.30**	**5.07**
Maternal depression or anxiety, 32 gestational weeks	1.48	0.91	0.1081	-0.34	3.31
**High social support, 8 weeks postpartum**	**-1.53**	**0.74**	**0.0433**	**-3.02**	**-0.05**
Gestational age (weeks)	-0.37	0.17	0.0360	-0.71	-0.02
Maternal obesity before pregnancy	-1.38	1.13	0.2265	-3.64	0.88
Maternal iron supplements during pregnancy	0.41	0.56	0.4651	-0.70	1.52

Note. Bolded values indicate a nominally significant association between a maternal psychosocial factor and NNL widths, after accounting for covariates as well as two other psychosocial factors tested.

© 2021 Mountain RV et al. *JAMA Network Open.*

eFigure 1. Correlations between measures of maternal psychosocial factors.

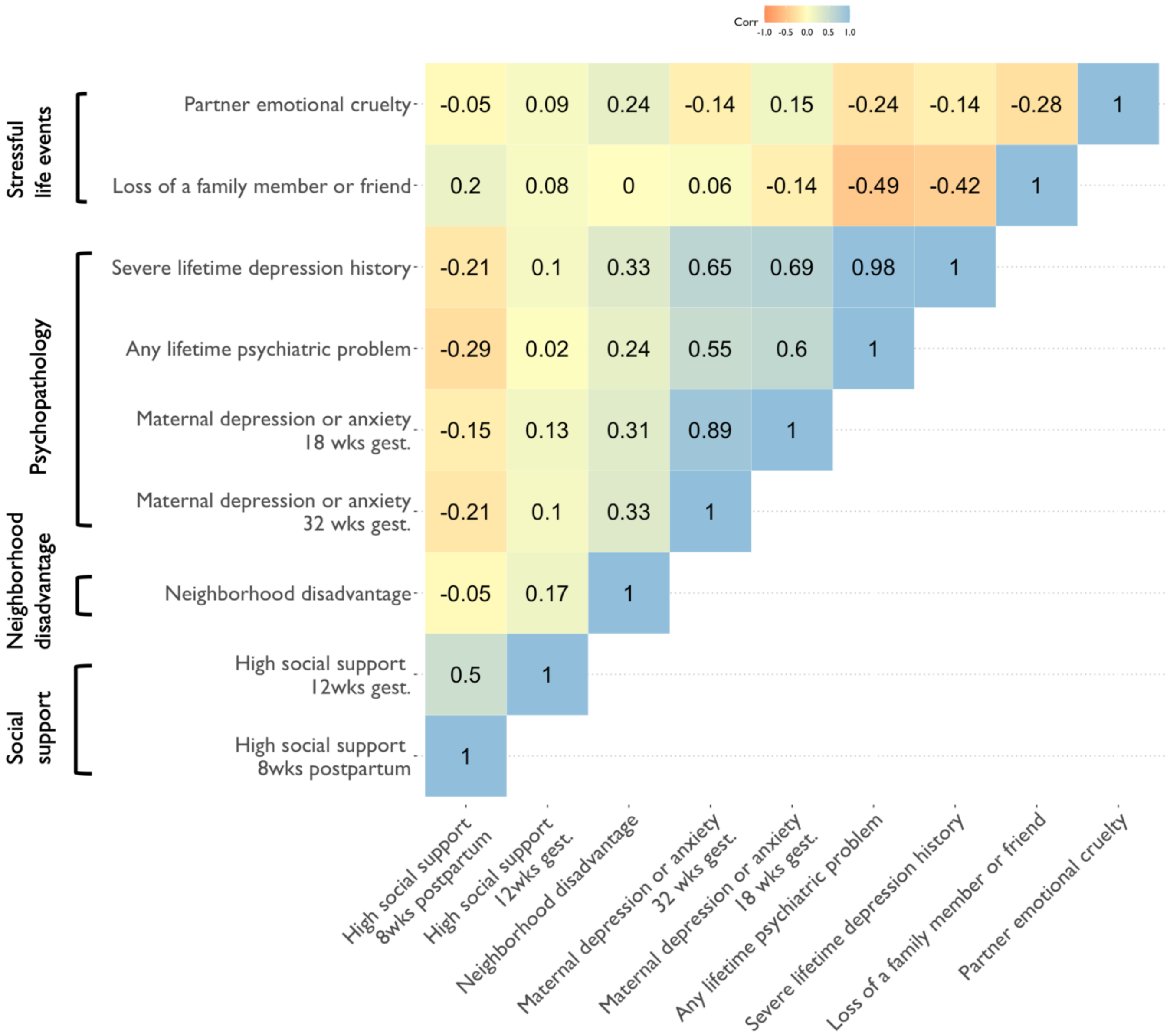

Note: Pairwise tetrachoric correlations are indicated in each cell to show the strength of correlations among measuers of psychosocial factors in the analytic sample. Because there is currently no established method to generate p-values for tetrachoric correlations, we did not report statistical significance.

© 2021 Mountain RV et al. *JAMA Network Open.*

eFigure 2. Distributions of neonatal line width measures.

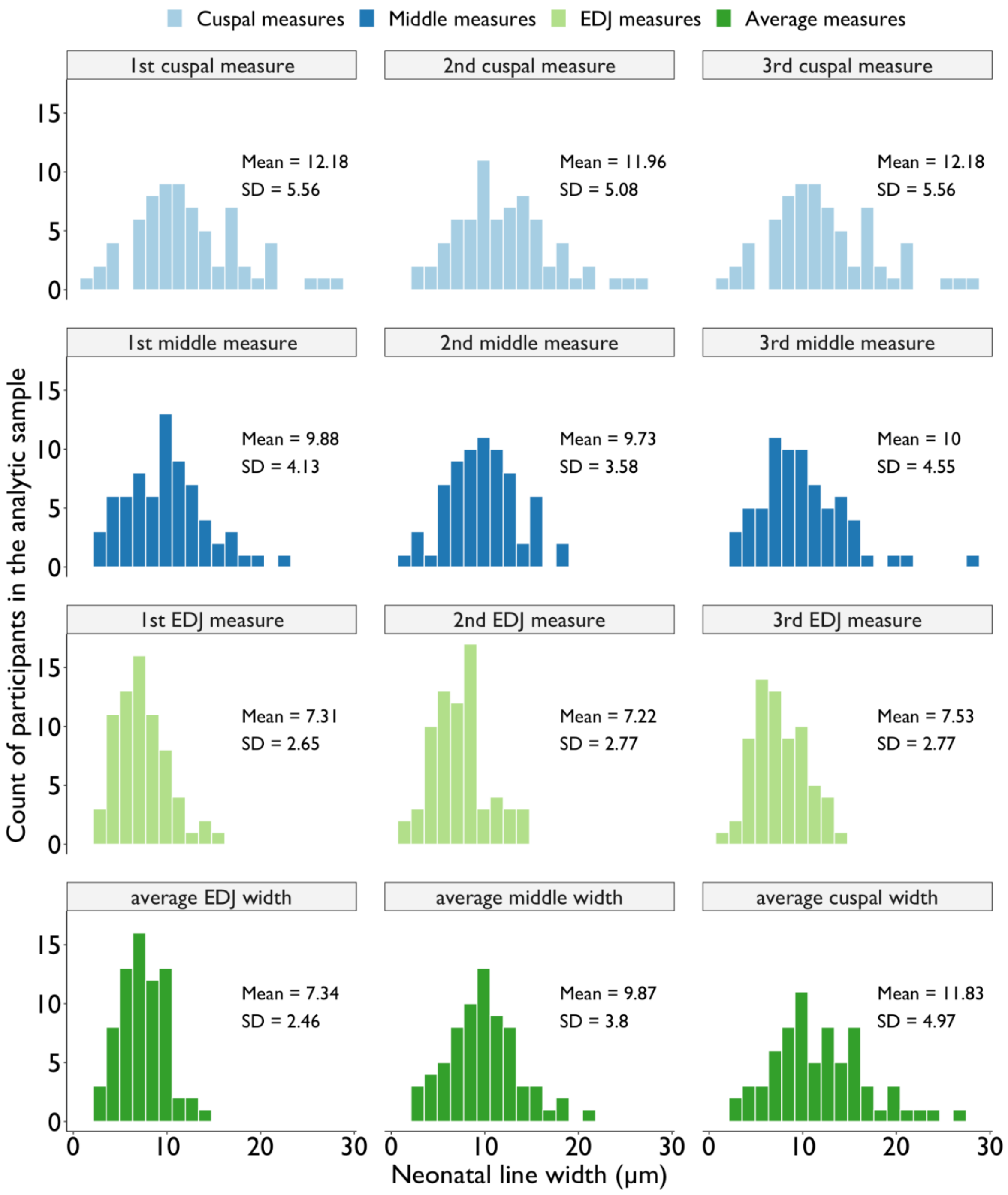

© 2021 Mountain RV et al. *JAMA Network Open.*

eFigure 3. Correlations between measures of neonatal line width.

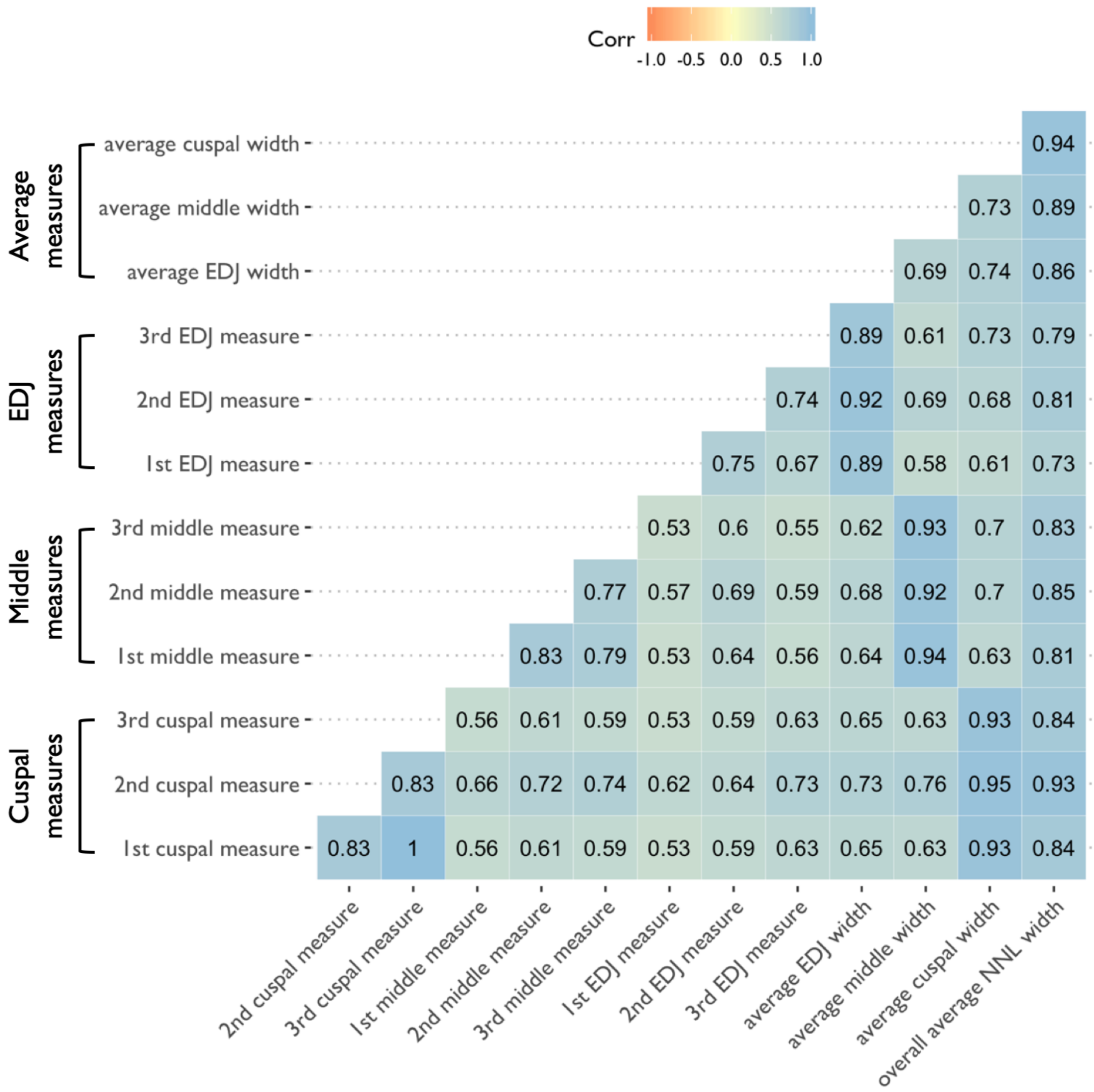

© 2021 Mountain RV et al. *JAMA Network Open.*

eFigure 4. Tooth position and the neonatal line width across individuals.

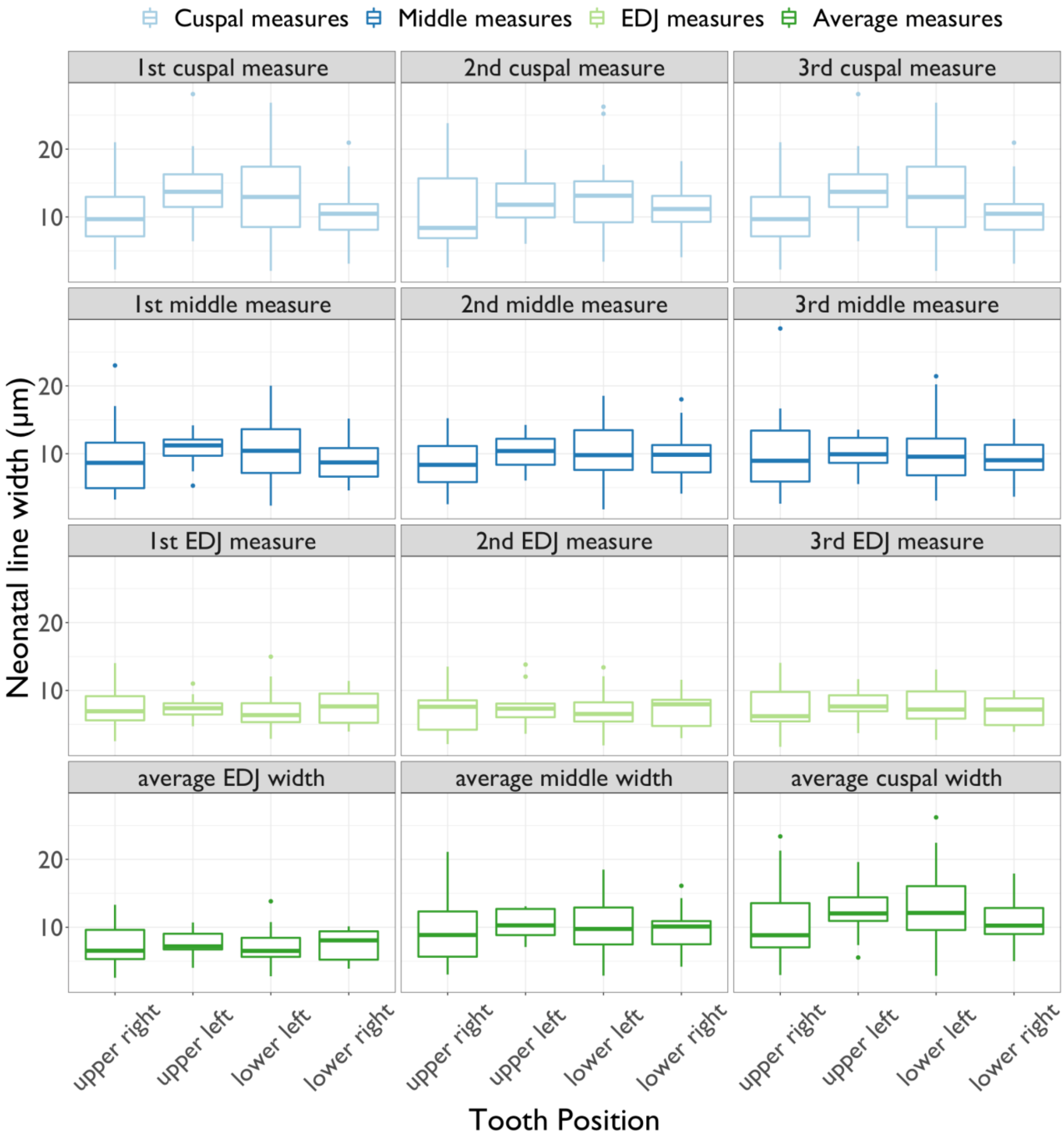

© 2021 Mountain RV et al. *JAMA Network Open.*

eFigure 5. *A priori* power analysis.

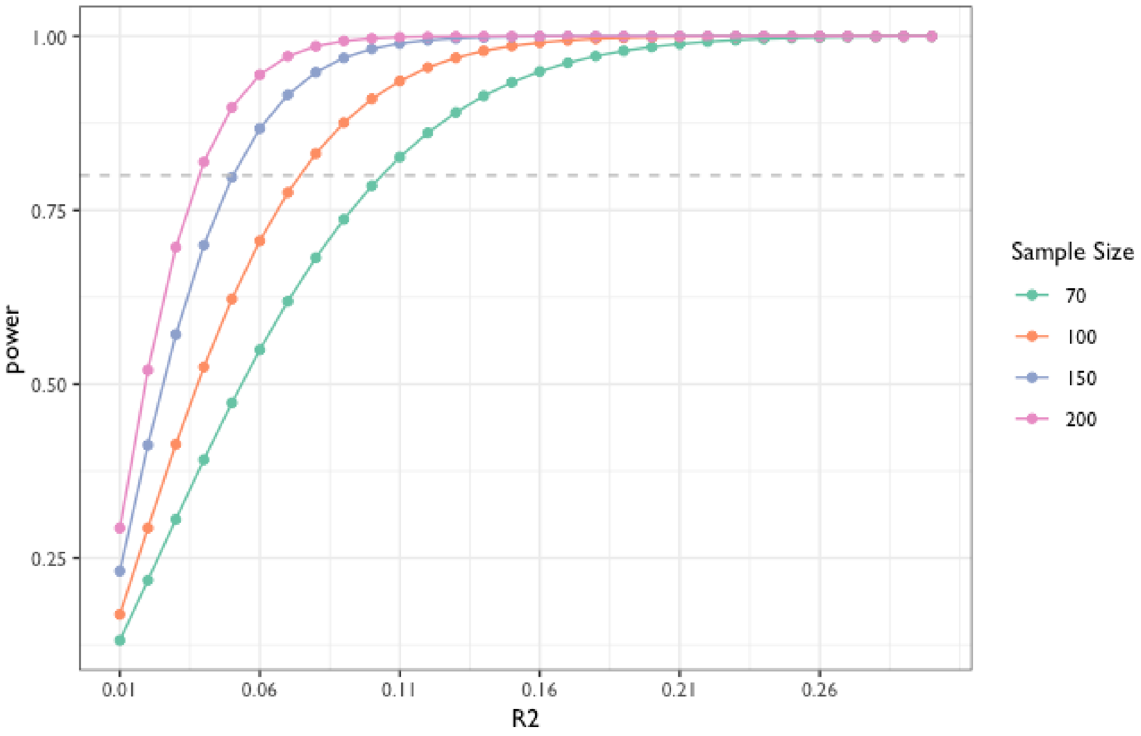

© 2021 Mountain RV et al. *JAMA Network Open*.

eFigure 6. Comparison of associations between maternal psychosocial factors during pregnancy and neonatal line widths before and after controlling for maternal polygenic risk score (PRS) for major depressive disorder.

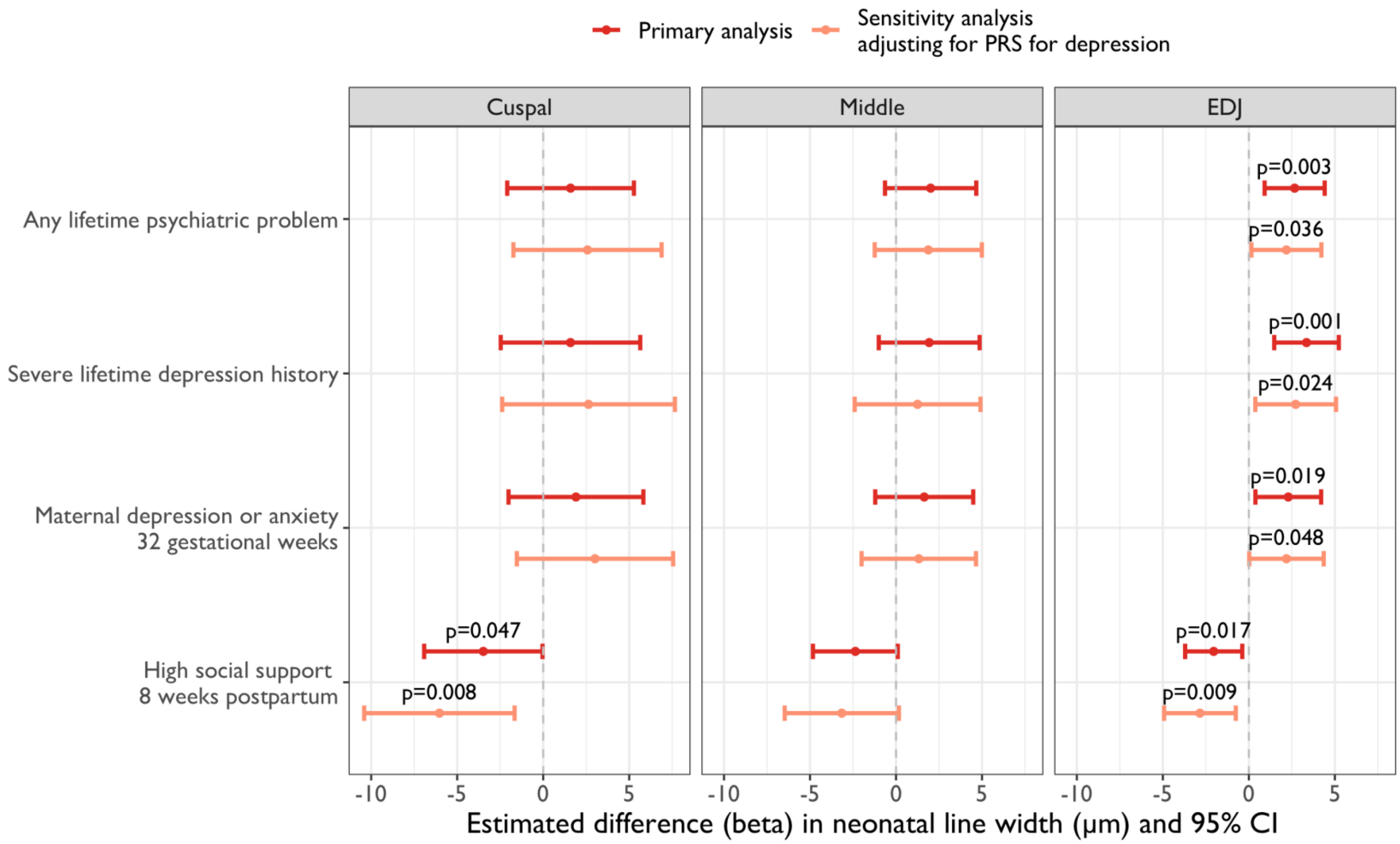

Note. The primary analysis was performed using the full analytic sample (n=70). The sensitivity analysis adjusting for maternal PRS for major depressive disorder was performed using a subset of the sample with available maternal genotype data (n=54). All covariates in the primary analysis were also adjusted for in the PRS sensitivity analysis.

© 2021 Mountain RV et al. *JAMA Network Open.*

eReferences

1 Golding, J., Pembrey, M., Jones, R. & The ALSPAC Study Team. ALSPAC: The Avon Longitudinal Study of Parents and Children I. Study methodology. *Paediatric and Perinatal Epidemiology* **15**, 74-87 (2001).

2 Boyd, A. *et al.* Cohort Profile: The 'Children of the 90s'—the index offspring of the Avon Longitudinal Study of Parents and Children. *International Journal of Epidemiology* **42**, 111-127, doi:10.1093/ije/dys064 (2013).

3 Hassett, B. R. *et al.* Effects of maternal, gestational, and perinatal variables on neonatal line width observed in a modern UK birth cohort. *American Journal of Physical Anthropology* **172**, 314-332, doi:10.1002/ajpa.24042 (2020).

4 Roberts, A. L., Lyall, K., Rich-Edwards, J. W., Ascherio, A. & Weisskopf, M. G. Maternal exposure to intimate partner abuse before birth is associated with autism spectrum disorder in offspring. *Autism* **20**, 26-36 (2016).

5 Janssen, P. A. *et al.* Intimate partner violence and adverse pregnancy outcomes: a population-based study. *American journal of obstetrics and gynecology* **188**, 1341-1347 (2003).

6 László, K. D. *et al.* Maternal bereavement during pregnancy and the risk of stillbirth: a nationwide cohort study in Sweden. *American journal of epidemiology* **177**, 219-227 (2013).

7 Birtchnell, J., Evans, C. & Kennard, J. The total score of the Crown-Crisp Experiential Index: a useful and valid measure or psychoneurotic pathology. *Br J Med Psychol* **61**, 255-266 (1988).

8 Crown, S. & Crisp, A. H. *Manual of the Crown-Crisp Experimental Index*. (Hodder & Stoughton, 1979).

9 Cox, J. L., Holden, J. M. & Sagovsky, R. Detection of postnatal depression. Development of the 10-item Edinburgh Postnatal Depression Scale. *Br J Psychiatry* **150**, 782-786, doi:10.1192/bjp.150.6.782 (1987).

10 Enoch, M.-A., Steer, C. D., Newman, T. K., Gibson, N. & Goldman, D. Early Life Stress, MAOA, and Gene-Environment Interactions Predict Behavioral Disinhibition in Children. *Genes, brain, and behavior* **9**, 65-74, doi:10.1111/j.1601-183X.2009.00535.x (2010).

11 Dunn, E. C. *et al.* What life course theoretical models best explain the relationship between exposure to childhood adversity and psychopathology symptoms: recency, accumulation, or sensitive periods? *Psychological Medicine* **48**, 2562-2572, doi:10.1017/s0033291718000181 (2018).

12 Baker, D., Taylor, H. & The Alspac Survey Team, H. The relationship between condition-specific morbidity, social support and material deprivation in pregnancy and early motherhood. *Social Science & Medicine* **45**, 1325-1336, doi:10.1016/S0277-9536(97)00059-2 (1997).

13 Skinner, M. & Dupras, T. Variation in birth timing and location of the neonatal line in human enamel. *Journal of forensic sciences* **38**, 1383-1390 (1993).

14 Kurek, M. *et al.* Prenatal factors associated with the neonatal line thickness in human deciduous incisors. *Homo : internationale Zeitschrift fur die vergleichende Forschung am Menschen* **66**, 251-263, doi:10.1016/j.jchb.2014.11.001 (2015).

15 Canturk, N., Atsu, S. S., Aka, P. S. & Dagalp, R. Neonatal line on fetus and infant teeth: An indicator of live birth and mode of delivery. *Early Hum Dev* **90**, 393-397, doi:10.1016/j.earlhumdev.2014.05.002 (2014).

16 Kurek, M. *et al.* Neonatal line width in deciduous incisors from Neolithic, mediaeval and modern skeletal samples from north-central Poland. *Annals of anatomy = Anatomischer Anzeiger : official organ of the Anatomische Gesellschaft* **203**, 12-18, doi:10.1016/j.aanat.2015.02.006 (2016).

17 Zanolli, C., Bondioli, L., Manni, F., Rossi, P. & Macchiarelli, R. Gestation length, mode of delivery, and neonatal line-thickness variation. *Hum Biol* **83**, 695-713, doi:10.3378/027.083.0603 (2011).

18 Eli, I., Sarnat, H. & Talmi, E. Effect of the birth process on the neonatal line in primary tooth enamel. *Pediatric dentistry* **11**, 220 (1989).

19 Noren, J. G. Microscopic study of enamel defects in deciduous teeth of infants of diabetic mothers. *Acta Odontologica Scandinavica* **42**, 153-156 (1984).

20 Noren, J., Grahnen, H. & Magnusson, B. Maternal diabetes and changes in the hard tissues of primary teeth: III. A histologic and microradiographic study. *Acta Odontologica Scandinavica* **36**, 127-135 (1978).

21 Behie, A. M. & Miszkiewicz, J. J. Enamel neonatal line thickness in deciduous teeth of Australian children from known maternal health and pregnancy conditions. *Early Human Development* **137**, 104821, doi:10.1016/j.earlhumdev.2019.07.004 (2019).

22 Huang, G. *et al.* Sex Differences in the Prenatal Programming of Adult Metabolic Syndrome by Maternal Androgens. *The Journal of Clinical Endocrinology & Metabolism* **103**, 3945-3953 (2018).

© 2021 Mountain RV et al. *JAMA Network Open.*

23 Fraser, A. *et al.* Cohort Profile: the Avon Longitudinal Study of Parents and Children: ALSPAC mothers cohort. *Int J Epidemiol* **42**, 97-110, doi:10.1093/ije/dys066 (2013).
24 Genomes Project, C. *et al.* An integrated map of genetic variation from 1,092 human genomes. *Nature* **491**, 56-65, doi:10.1038/nature11632 (2012).
25 Marchini, J., Howie, B., Myers, S., McVean, G. & Donnelly, P. A new multipoint method for genome-wide association studies by imputation of genotypes. *Nature Genetics* **39**, 906, doi:10.1038/ng2088 https://www.nature.com/articles/ng2088#supplementary-information (2007).
26 Purcell, S. *et al.* PLINK: a toolset for whole-genome association and population-based linkage analysis. *American Journal of Human Genetics* **81**, 559-575 (2007).
27 Wray, N. R. *et al.* Genome-wide association analyses identify 44 risk variants and refine the genetic architecture of major depression. *Nature genetics* **50**, 668, doi:10.1038/s41588-018-0090-3 (2018).
28 Howard, D. M. *et al.* Genome-wide meta-analysis of depression identifies 102 independent variants and highlights the importance of the prefrontal brain regions. *Nat Neurosci*, doi:10.1038/s41593-018-0326-7 (2019).

© 2021 Mountain RV et al. *JAMA Network Open.*
